# Metabolic clearance of oxaloacetate and mitochondrial complex II respiration: Divergent control in skeletal muscle and brown adipose tissue

**DOI:** 10.1016/j.bbabio.2022.148930

**Published:** 2022-10-19

**Authors:** Liping Yu, Brian D. Fink, Ritu Som, Adam J. Rauckhorst, Eric B. Taylor, William I. Sivitz

**Affiliations:** a Department of Biochemistry and Molecular Biology, University of Iowa, Iowa City, IA 52242, USA; b Carver College of Medicine NMR Core Facility, University of Iowa, Iowa City, IA 52242, USA; c Department of Internal Medicine/Endocrinology and Metabolism, University of Iowa and the Iowa City Veterans Affairs Medical Center, Iowa City, IA 52242, USA; d Department of Molecular Physiology and Biophysics, University of Iowa, Iowa City, IA 52242, USA

**Keywords:** Oxaloacetate, Succinate dehydrogenase, Mitochondria, Brown adipose tissue, Muscle, Mitochondrial complex II

## Abstract

At low inner mitochondrial membrane potential (ΔΨ) oxaloacetate (OAA) accumulates in the organelles concurrently with decreased complex II-energized respiration. This is consistent with ΔΨ-dependent OAA inhibition of succinate dehydrogenase. To assess the metabolic importance of this process, we tested the hypothesis that perturbing metabolic clearance of OAA in complex II-energized mitochondria would alter O_2_ flux and, further, that this would occur in both ΔΨ and tissue-dependent fashion. We carried out respiratory and metabolite studies in skeletal muscle and interscapular brown adipose tissue (IBAT) directed at the effect of OAA transamination to aspartate (catalyzed by the mitochondrial form of glutamic-oxaloacetic transaminase, Got2) on complex II-energized respiration. Addition of low amounts of glutamate to succinate-energized mitochondria at low ΔΨ increased complex II (succinate)-energized respiration in muscle but had little effect in IBAT mitochondria. The transaminase inhibitor, aminooxyacetic acid, increased OAA concentrations and impaired succinate-energized respiration in muscle but not IBAT mitochondria at low but not high ΔΨ. Immunoblotting revealed that Got2 expression was far greater in muscle than IBAT mitochondria. Because we incidentally observed metabolism of OAA to pyruvate in IBAT mitochondria, more so than in muscle mitochondria, we also examined the expression of mitochondrial oxaloacetate decarboxylase (ODX). ODX was detected only in IBAT mitochondria. In summary, at low but not high ΔΨ, mitochondrial transamination clears OAA preventing loss of complex II respiration: a process far more active in muscle than IBAT mitochondria. We also provide evidence that OAA decarboxylation clears OAA to pyruvate in IBAT mitochondria.

## 1. Introduction

Most electron entry into mitochondria occurs at complex I or complex II when energized by NADH or succinate, respectively. Complex I may reside largely in contact with complexes III and IV in the form of respirasomes while complex II exists as a much smaller singular structure [1,2]. Nonetheless, when energized by succinate in the absence of downstream constraints, thermodynamic considerations [3] imply that electron donation by succinate can generate an inner membrane potential (ΔΨ)^1^ even greater than that for NADH. Recent attention has been directed to the concept that regulation of succinate oxidation by succinate dehydrogenase (SDH, or complex II) may be an important means to adjust mitochondrial function according to the need or lack of need for overall cell energy [3-6].

One important way by which SDH activity can be regulated is through inhibition by the downstream metabolite, oxaloacetate (OAA). Although, this effect of OAA is long known [7-13], its significance and underlying mechanism have received relatively little attention. We believe this is due in part to the instability of OAA, making it very difficult to detect by mass spectrometry or other means. Moreover, studies of complex II-energized respiration have classically been performed by energizing mitochondria with succinate along with rotenone to block electron flow through complex I. However, rotenone also blocks malate conversion to OAA, thus obscuring any feedback effect of OAA to inhibit complex II. Further, to control the effect of ΔΨ on OAA production and O_2_ flux under phosphorylating conditions, it is necessary to maintain a steady level of potential. This is often not done in studies of isolated mitochondrial respiration. More often, a given amount of ADP is administered after which ΔΨ decreases but then increases as ADP is continuously consumed by ATP synthase.

In past studies of mouse mitochondria, we provided compelling evidence that the effect of OAA to inhibit complex II respiration in skeletal muscle and interscapular brown adipose tissue (IBAT) is strongly dependent on mitochondrial inner membrane potential [14-16]. To demonstrate this, we carried out studies of succinate-energized O_2_ flux under conditions wherein ADP concentrations, and consequently ΔΨ, are clamped by addition of 2-deoxyglucose (2DOG) plus hexokinase, enabling measurement of respiration at given constant levels of membrane potential. In addition, we used a highly specific 2-dimensional (2D) NMR method that we developed [17,18] using ^13^C-labeled TCA substrates to determine OAA concentrations. We found that when succinate-energized muscle mitochondria were titrated with ADP, respiration initially increased as ATP production consumed ΔΨ. However, at a certain point, although ΔΨ continued to drop, OAA accumulated, thereby inhibiting SDH and decreasing respiration. Further, we observed a similar increasing then decreasing rate of O_2_ flux when mitochondria were titrated with the chemical uncoupler carbonyl cyanide p-trifluoromethoxy-phenylhydrazone (FCCP). Moreover, we found similar ΔΨ-dependent changes in O_2_ flux in succinate-energized IBAT mitochondria by modulating potential with GDP [14,15], a well-recognized potent inhibitor of uncoupling protein 1 (UCP1) [19].

We carried out metabolite and redox studies to address the mechanism underlying the above phenomenon. Our results implied that the mechanism involved a sequence of events triggered by perturbed ΔΨ. The sequence starts with the effects of ΔΨ on succinate-driven reverse electron transport (RET) to complex I [15,16], a phenomenon strongly favored by high ΔΨ [20,21] and known to increase the NADH/NAD^+^ ratio [22,23]. We found that in succinate-energized mitochondria at high ΔΨ and high RET, the NADH/NAD^+^ ratio was maintained towards the reduced state, thus reducing malate conversion to OAA and enabling unimpaired SDH activity [14,16,18]. However, at low ΔΨ, and therefore low RET, the NADH/NAD^+^ ratio decreased associated with increased [OAA] and decreased respiration. Of note is that the malate dehydrogenase (MDH) reaction under in vitro equilibrium conditions, is far to the left [24]. However, as we and others [5,15,16] have shown, only small amounts of OAA within the range of what we detected in our past work [14,16] are needed to inhibit SDH. Hence, any rightward change would be important.

Importantly, we showed that the drop in O_2_ flux at low ΔΨ in either muscle or IBAT mitochondria can be completely and rapidly reversed by addition of the complex I substrate pyruvate, by addition of rotenone to maintain NADH in the reduced state, or by *N*,*N*,*N′*,*N′*-Tetramethyl-*p*-phenylenediamine dihydrochloride (TMPD) to provide electrons directly to complex IV [15,16,18], thus proving that the loss of succinate-energized respiration is not related to time of incubation or damage to mitochondria.

Given the role of OAA in regulating energy needs, more understanding is needed regarding the control of mitochondrial OAA concentrations. The above-mentioned past studies have helped understand the mechanism and ΔΨ-dependent nature of OAA to inhibit SDH. However, whether mitochondrial metabolism of OAA affects mitochondrial function has not been determined.

In the work reported here, we hypothesized that altering the metabolic clearance of OAA in mouse mitochondria would alter complex II-energized mitochondrial respiration and, moreover, that this would occur in a ΔΨ-dependent fashion. We focused on the role of mitochondrial transamination to clear OAA to aspartate catalyzed by the mitochondrial form of glutamic-oxaloacetic transaminase (Got2). Data from the Human Protein Atlas, Mouse Genome Informatics, and National Center for Biotechnology Information (NCBI) suggest greater expression of Got2 in muscle than adipocytes (although brown adipocyte data is lacking). Therefore, we further hypothesized that the effect of Got2 mediated-OAA clearance on O_2_ flux would manifest in both a ΔΨ and tissue-dependent fashion. In this work, we studied metabolite flow by NMR and mass spectrometry, mitochondrial O_2_ flux and potential, and gene expression in skeletal muscle and IBAT mitochondria to address the effect of OAA metabolism to aspartate on complex II respiration. Beyond conversion of OAA to aspartate, our metabolite studies incidentally found evidence for conversion of OAA to pyruvate in IBAT mitochondria. Therefore, we also examined the expression of mitochondrial oxaloacetate decarboxylase (ODX, also known as FAHD1), which was reported identical to one of the fumarylacetoacetate hydrolase domain-containing proteins and present in mammalian tissues [25].

## 2. Materials and methods

### 2.1. Reagents and supplies

GDP, ADP, 2-deoxyglucose, [U—^13^C]-succinate, and [U-^13^C]-malate were obtained from Millipore Sigma, Burlington, MA. Otherwise, reagents, kits, and supplies were as specified or purchased from standard sources.

### 2.2. Animal procedures

Animals were maintained according to National Institute of Health guidelines and the protocol was approved by our Institutional Animal Care and Use Committee. Male C57BL/6J mice (Jackson Laboratories, Bar Harbor, Maine) were fed a normal rodent diet (diet 7001, Teklad, Envigo, Indianapolis, IN) until sacrifice at age 6 to 10 weeks. Mice were euthanized by isoflurane overdose and cardiac puncture.

### 2.3. Preparation of mitochondria

Mitochondria were prepared by differential centrifugation and purified using a Percoll gradient as we have described [26]. Mitochondrial integrity was assessed by cytochrome C release using a commercial kit (Cytochrome C Oxidase Assay Kit, Millipore-Sigma, St. Louis), indicating a mean of 96 % intact mitochondria, an acceptable range compared to mitochondrial preparations from several sources [27].

### 2.4. Respiration and membrane potential

All studies of mitochondrial respiration and inner membrane potential utilized freshly isolated and purified mitochondria on the day of the experiments. O_2_ flux was determined using an Oxygraph-2 k high resolution respirometer (Oroboros Instruments, Innsbruck, Austria). When measured, ΔΨ was determined simultaneously with respiration using a potential sensitive tetraphenylphosphonium (TPP^+^) electrode fitted into the Oxygraph incubation chamber with a volume of 2 ml. A TPP^+^ standard curve was performed in each run by adding tetraphenylphosphonium chloride at concentrations of 0.25, 0.5, and 0.75 μM prior to the addition of mitochondria to the chamber. Different amounts of mitochondria were required for the respirometry (Oxygraph) studies depending on substrate conditions and whether the organelles were subsequently processed for determination of metabolites by NMR or mass spectrometry. We used 0.1 mg/ml for mitochondrial incubations energized by the various substrates (except malate, see below) with the media not further processed for NMR or LC-MS. A larger amount (0.35 mg/ml) of mitochondria were used for experiments wherein the Oxygraph media was subsequently processed for NMR or LC-MS. Mitochondrial incubations energized by malate required 0.6 mg mitochondria/ml due to lower rates of respiration. Mitochondria were incubated at 37 °C in 2 ml of ionic respiratory buffer (105 mM KCl, 10 mM NaCl, 5 mM Na_2_HPO_4_, 2 mM MgCl_2_, 10 mM HEPES pH 7.2, 1 mM EGTA, 0.2 % defatted BSA) with 10 U/ml hexokinase (Worthington Biochemical), and 10 mM 2-deoxyglucose (2DOG). When ADP was included in incubations, the concentrations were clamped (see below) at the desired level.

Although the O_2_ tension in the Oxygraph drops with time, the rate of respiration is little affected until levels become very low. However, since incubations were carried out for 20 min, it was necessary to open the chamber at certain points to prevent marked deterioration in the oxygen content of the medium. A representative Oxygraph tracing is shown in Supplemental Fig. S1.

### 2.5. ADP recycling and generation of the 2-deoxyglucose ATP energy clamp

This was done using a method we previously [26,28] developed to carry out studies of isolated mitochondria under conditions of clamped ADP and membrane potential. Mitochondrial incubations were performed in the presence of hexokinase, excess 2-deoxyglucose (2DOG), and varying amounts of added ADP. ATP so generated under these conditions drives the conversion of 2DOG to 2DOG phosphate (2DOGP) while regenerating ADP. The reaction occurs rapidly and irreversibly thereby effectively clamping membrane potential determined by available ADP. This was in fact the case as we have demonstrated in the past for rat and mouse muscle [16,28], mouse liver [29], and mouse heart mitochondria [29].

### 2.6. Metabolite measurements by NMR

Metabolite measurements were performed as we previously described [16-18] on the contents of the Oxygraph chamber after mitochondrial incubation with ^13^C-labeled substrates for 20 min in the same media used for measuring respiration. Immediately after mitochondrial incubations, 1.5 ml of the chamber content was placed in tubes on ice and acidified with 91 μl of 70 % perchloric acid. The solutions were then thoroughly mixed, sonicated on ice for 30 s at a power setting of 4 W, and stored at −80 °C for up to 2 weeks. The sample tubes were then thawed on ice and centrifuged at 50,000 ×*g* for 20 min at 4 °C. Supernatants were removed and 10 N KOH was added to bring the pH to 7.4 followed by centrifugation at 16,000 ×*g* for 15 min at 4 °C to remove precipitated salts. The cleared, neutralized supernatants were then stored at −80 °C prior to NMR studies. For NMR sample preparations, 350 μl of the stored supernatant was added to 150 μl of 50 mM sodium phosphate, pH 7.4 in deuterium oxide for metabolite measurement. ^13^C and ^3^H NMR assignments of succinate, malate, fumarate, oxaloacetate (OAA), citrate, pyruvate, aspartate, glutamate, and α-ketoglutarate were obtained by using standard compounds. OAA was found to be unstable with a half-life about 14 h when tested at pH 7.4 and temperature at 25 °C. Therefore, after mitochondrial incubation, perchloric acid extraction was carried out as quickly as possible to destroy the mitochondrial enzymes and minimize the degradation of OAA. In addition, for determination of stability, known amounts of OAA were subjected to parallel incubation, perchloric acid extraction, neutralization, and storage.

We examined the effect of sample storage under the acidified condition for oxaloacetate, malate, fumarate, α-ketoglutarate, and pyruvate. No loss of stability was found over one- or two-week time periods (Supplemental Fig. S2). Succinate and citrate should be stable when acidified based on their structure. Moreover, even if there were some small effects of storage, that should be equal for samples comparing one condition to another (as samples from each condition for a given experiment were stored side by side over the same time period).

Both ^13^C/^1^H HSQC and HMQC spectra were collected at 25 °C on a Bruker Avance II 800 MHz NMR spectrometer equipped with a sensitive cryoprobe for the perchloric acid-extracted samples for quantification of metabolites of the mitochondrial incubations. All NMR spectra were processed using NMRPipe package [30] and analyzed using NMRView [31]. Peak heights were used for quantification.

Of note, the metabolite concentrations measured here were determined on respiratory medium after disruption of mitochondria. For the added substrates, ^13^C-succinate and ^13^C-glutamate, these values largely reflect additions to the medium. All other metabolites could only have accumulated from mitochondrial metabolism. This is because the 2D NMR technique measures only ^13^C-labeled metabolites generated from the added ^13^C-substrates and does not reflect the presence or metabolism of any unlabeled compounds that might have been present in our mitochondria after isolation.

### 2.7. Liquid chromatography mass spectrometry (LC-MS) and [^13^C] isotopomer flux analysis

For *flux analysis,* LC-MS was utilized to determine ^13^C-isotopologue enrichments of mitochondrial metabolites following incubation with uniformly labeled [U-^13^C]-succinate + unlabeled glutamate. Reactions were carried out in the Oxygraph 2 k respirometer for 20 min. Chamber contents were transferred to 2 ml tubes and immediately snap frozen in liquid nitrogen. The frozen samples were lyophilized overnight and extracted in ice cold 2:2:1 acetonitrile:methanol:water containing D8-valine as an internal standard. Crude extracts were centrifuged to remove insoluble material, dried using a SpeedVac vacuum concentrator, and resuspended to a 10× concentrate based on the dried extract's original volume using 1:1 acetonitrile:water for LC-MS analysis.

2 μl of the prepared samples were separated using a Millipore SeQuant ZIC-pHILIC (2.1 × 150 mm, 5 μm particle size) column with a ZIC-pHILIC guard column (20 × 2.1 mm) attached to a Thermo Vanquish Flex UHPLC. Mobile phase comprised Buffer A [20 mM (NH_4_)_2_CO_3_, 0.1 % NH_4_OH] and Buffer B [acetonitrile]. The chromatographic gradient was run at a flow rate of 0.150 ml/min as follows: 0–21 min-linear gradient from 80 to 20 % Buffer B; 20–20.5 min-linear gradient from 20 to 80 % Buffer B; and 20.5–28 min-hold at 80 % Buffer B. Data was acquired using a Thermo Q Exactive MS operated in negative polarity targeted selected ion monitoring (tSIM) mode with a spray voltage set to 3.0 kV, the heated capillary held at 275 °C, and the HESI probe held at 350 °C. The sheath gas flow was set to 40 units, the auxiliary gas flow was set to 15 units, and the sweep gas flow was set to 1 unit. MS data resolution was set at 70,000, the AGC target at 10e^6^, and the maximum injection time at 200 ms. The mass isolation window was set to 12 *m/z* and the isolation offset to 5 allowing for the observation of −1 to +11 m/z of each metabolite targeted. The tSIM inclusion list was populated using target metabolites chemical formulas and their corresponding retention times previously determined using neat standards.

In other experiments, we incubated mitochondria with unlabeled malate (5 mM) plus unlabeled L-glutamate (0.5 mM) for 20 min prior to harvest by snap freezing in liquid N_2_. Each sample tube contained 1.2 mg of mitochondria in a 2 ml volume of ionic respiration medium. Frozen samples were lyophilized overnight and extracted in ice cold 2:2:1 acetonitrile:methanol:water containing D4-succinate as an internal standard. Crude extracts were centrifuged to removed insoluble material and cleared supernatants were transferred to new autosampler vials for flow injection analysis. 2 μl of the cleared extracts were injected into 1:1 Buffer A:Buffer B as described above at a flow rate of 0.1 ml/min. Data was acquired using a Thermo Q Exactive MS operating as described above with the following modifications: resolution was set at 35,000, mass isolation window was set to 1.5 *m/z*, and the isolation offset was set to 0. The tSIM inclusion list was populated using target metabolites chemical formulas and data was collected from 0.1 to 4 min.

For both flux and flow injection analysis, LC-MS data were processed using the Thermo Scientific TraceFinder 4.1 and TraceFinder 5.1 software. Targeted metabolites were identified based on accurate masses and retention times referenced against the University of Iowa Metabolomics Core facility's database of in-house confirmed standards.

### 2.8. Immunoblot analyses

30 micrograms of protein per lane were separated on a precast gel (Ready Gel, Stain-Free, TGX 4–20 % 10-Well, BioRad, Hercules, CA) and electroblotted to nitrocellulose membranes (BioRad 0.45 μm). Blots were blocked with 5 % milk in Tris-buffered saline with 0.1 % Tween-20 (T-TBS) for 30 min and incubated overnight at 4 °C with affinity-purified antibody to Got2 (1/1000, rabbit polyclonal anti-GOT2, Millipore Sigma, Burlington, MA, #AV43517-100UL), Got1 (1/1000, rabbit polyclonal anti-GOT1, Proteintech, Rosemont, IL, # 14886–1-AP), ODX (also known as FAHD1, 1/500 rabbit anti-FAHD1 polyclonal, MyBioSource, San Diego, CA), Porin (1/2000 rabbit polyclonal ant-porin, Abcam, Cambridge, UK, ab154856), mitochondrial malic enzyme 2 (1/400 mouse monoclonal anti ME2, Santa Cruz, CA, # sc-514,850), or mitochondrial malic enzyme 3 (1/1000 rabbit monoclonal anti ME3, Abcam, Cambridge, UK, ab172972). Blots were washed with T-TBS and exposed to anti-rabbit horseradish peroxidase (HRP)-conjugated secondary antibody (1/10,000, Anti-rabbit IgG, HRP-linked Antibody, Cell Signaling Technology, Danvers, MA, #7074S) or anti-mouse HRP-linked secondary antibody (1/10,000, Anti-mouse IgG, Cell Signaling Technology, Danvers, MA, #7076S). Blots were washed again and developed by enhanced chemiluminescence using a standard kit (ECL prime, Amersham Pharmacia Biotech, Piscataway, NJ).

### 2.9. mRNA quantification

Quantitative PCR was performed using a ThermoFisher Scientific verso SYBR Green 1-step qRT-PCR low ROX mix Kit (Cat No. AB4106A) and an Applied Biosystems (Waltham, MA) 7500 Realtime PCR system. Amplification was performed at a dissociation temperature of 95 °C, annealing at 60 °C, and extension at 72 °C. Melt curves documented the specificity of the amplified products. Quantitation was carried out comparing CT values for genes of interest compared to amplified ribosomal protein lateral stalk subunit P0 (Rplp0) which proved highly consistent over repeated runs consistent with other reports [32,33]. Indeed, we found less variability with Rp1p0 compared to actin-β (Actb) which was also assessed. Primers were recommended by and purchased from Integrated DNA Technologies (Coralville, IA) and listed in Supplementary Table S1.

### 2.10. Statistics

Data were analyzed by unpaired *t*-test or one or two factor ANOVA with multiple comparisons as indicated in the figure legends using GraphPad Prism (GraphPad Software, Inc., La Jolla, CA). Significance was considered at *p* < 0.05.

## 3. Results

### 3.1. Rescue of succinate-energized respiration at low ΔΨ by pyruvate or glutamate

**Fig. 1.**
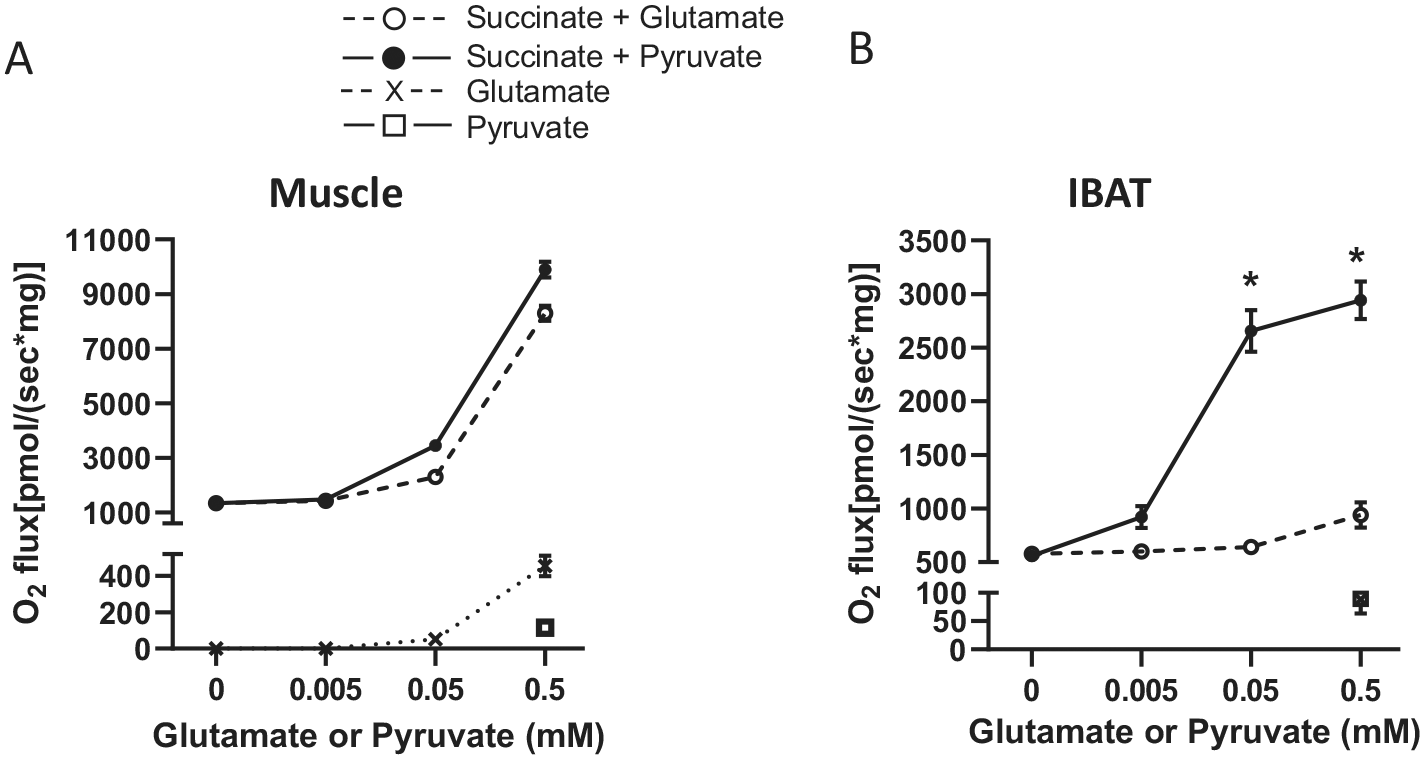
Glutamate or pyruvate titration of complex II (succinate)-energized mitochondrial respiration. A) Skeletal muscle mitochondria were energized with 10 mM succinate plus zero or low concentrations of glutamate or pyruvate as indicated on the x-axis. Each data point represents the mean O2 flux ± sem during individual 8-to-10-min incubations at low ΔΨ (ADP clamped at 32 μM). In addition, some incubations were performed with glutamate or pyruvate alone in the absence of succinate, *n* = 3 incubations for all data points. B) IBAT mitochondrial were incubated under the conditions of panel A except that ADP was not added since ΔΨ is intrinsically low due to UCP1. *n* = 5 for succinate + glutamate, *n* = 4 for succinate + pyruvate, n = 3 for glutamate or pyruvate in the absence of succinate. * *p* < 0.001 for pyruvate effect versus glutamate effect in the presence of succinate by two-way ANOVA with Sidak's method for multiple comparisons.

Fig. 1 demonstrates the effect of adding different amounts of pyruvate or glutamate to succinate-energized muscle or IBAT mitochondria at low ΔΨ. Low ΔΨ was induced by 32 μM ADP in muscle mitochondria or was intrinsically low due to UCP1 in IBAT mitochondria. As we previously showed [15,16], succinate energized O_2_ flux is low under these conditions when compared to O_2_ flux at higher ΔΨ. As shown in Fig. 1, either adding pyruvate to clear OAA to citrate (via acetyl-CoA from pyruvate) or adding glutamate to clear OAA to aspartate (via Got2) increases respiration in dose-dependent fashion. This occurs at concentrations of pyruvate or glutamate that, when added alone (i.e., without succinate), have minimal effects on O_2_ flux. Of note, the relative effects of pyruvate versus glutamate differed markedly between these two tissues, indicating far less capacity of glutamate to clear OAA in IBAT mitochondria.

### 3.2. Role of OAA transamination on O2 flux and ΔΨ in muscle mitochondria

**Fig. 2.**
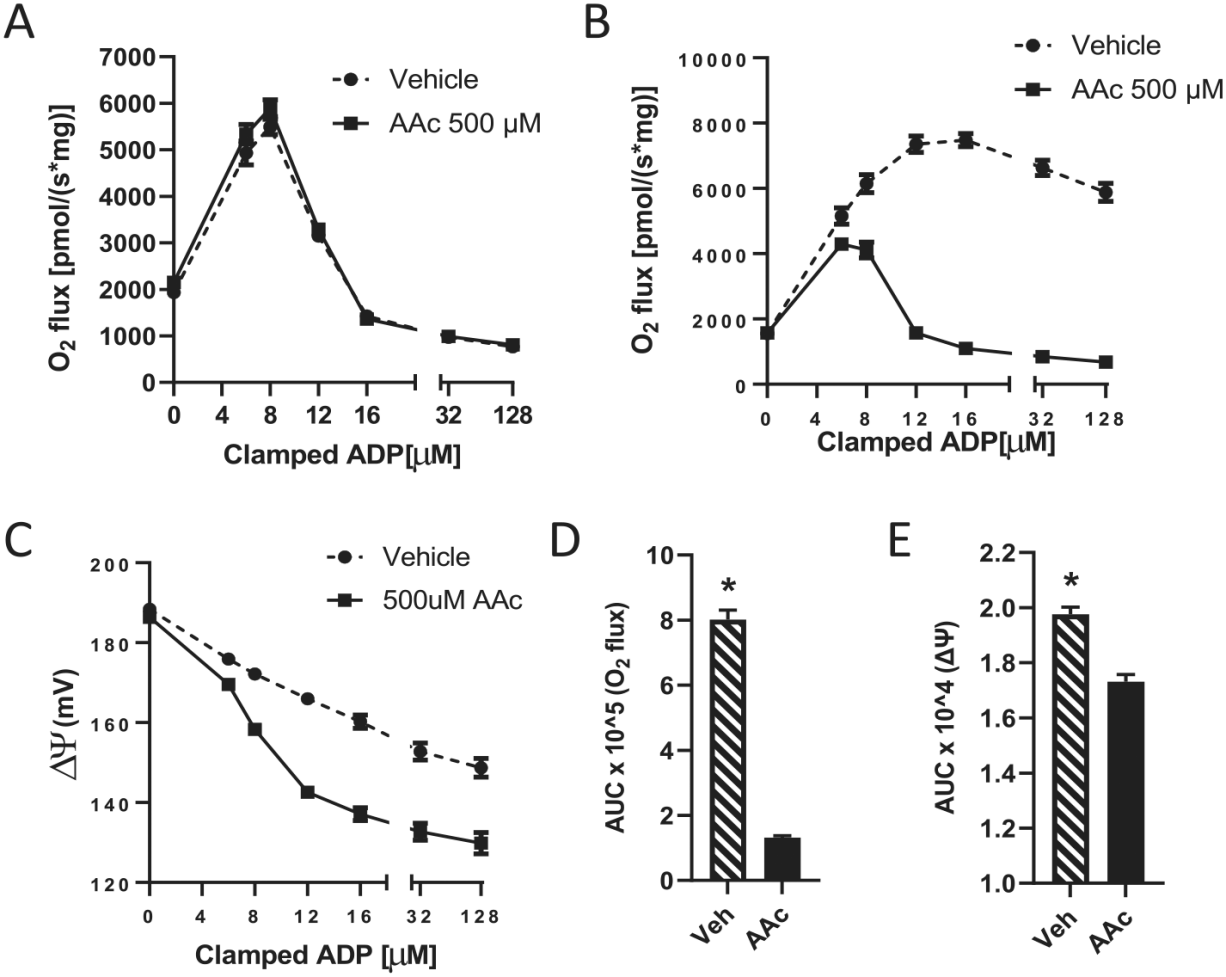
Effect of the transaminase inhibitor, aminooxyacetic acid (AAc) on O2 flux and ΔΨ in muscle mitochondria. A) O2 flux in mitochondria energized by 10 mM succinate alone with or without AAc and titrated with sequential clamped concentrations of ADP, *n* = 4. B) O2 flux in incubations carried out as in panel A energized by 10 mM succinate but with 0.5 mM glutamate added as a nitrogen source for Got2 catalyzed transamination of OAA, *n* = 9. C) Inner membrane potential corresponding to the respiratory measurements of panel B. D-E) Area under the curves of respiration and potential depicted in panels B and C (respectively). Data represent mean ± sem, * *p* < 0.0001 versus AAc by 2-tailed, unpaired *t*-test.

Fig. 2 shows the effect of inhibiting the mitochondrial enzyme, Got2, with the transaminase inhibitor, aminooxy acetic acid (AAc), on succinate-energized respiration and potential in muscle mitochondria. Mitochondria were titrated with ADP at clamped concentrations to manipulate ΔΨ. Fig. 2A depicts the effect of ADP titration in the absence of a nitrogen source for the transamination of OAA to aspartate and shows that ADP titration induced an initial rise followed by a decrease in O_2_ flux. This is consistent with what we reported in the past [16]. Addition of AAc had no effect under these conditions. When a low concentration of glutamate was added to enable the transamination of OAA to aspartate, the drop in respiration at high [ADP] was somewhat mitigated (compare Fig. 2A and B). However, addition of AAc completely prevented any such mitigation and markedly reduced respiration at ADP concentrations above 4 to 8 μM. Fig. 2C documents the drop in potential associated with the ADP additions of Fig. 2B. The decrease in ΔΨ is greater in the presence of AAc as expected since lower respiration (Fig. 2B) results in less proton pumping and hence, less membrane voltage differential. Fig. 2D-E quantitate the effects of AAc on O_2_ flux and ΔΨ as area under the curve.

In our past work, we showed that the impaired respiration of succinate-energized mitochondria at low ΔΨ (high ADP concentration as shown in Fig. 2A) can be cleared in seconds by adding pyruvate as a source of acetyl-CoA, proving that the low respiration at high [ADP] is not due to mitochondrial damage [15,18]. To prove that this is the case even in the presence of AAc, we performed additional incubations adding pyruvate at high [ADP] (Supplementary Fig. S3).

### 3.3. Inhibition of mitochondrial transaminase (Got2) alters metabolite accumulation and O2 flux in succinate-energized muscle mitochondria

**Fig. 3.**
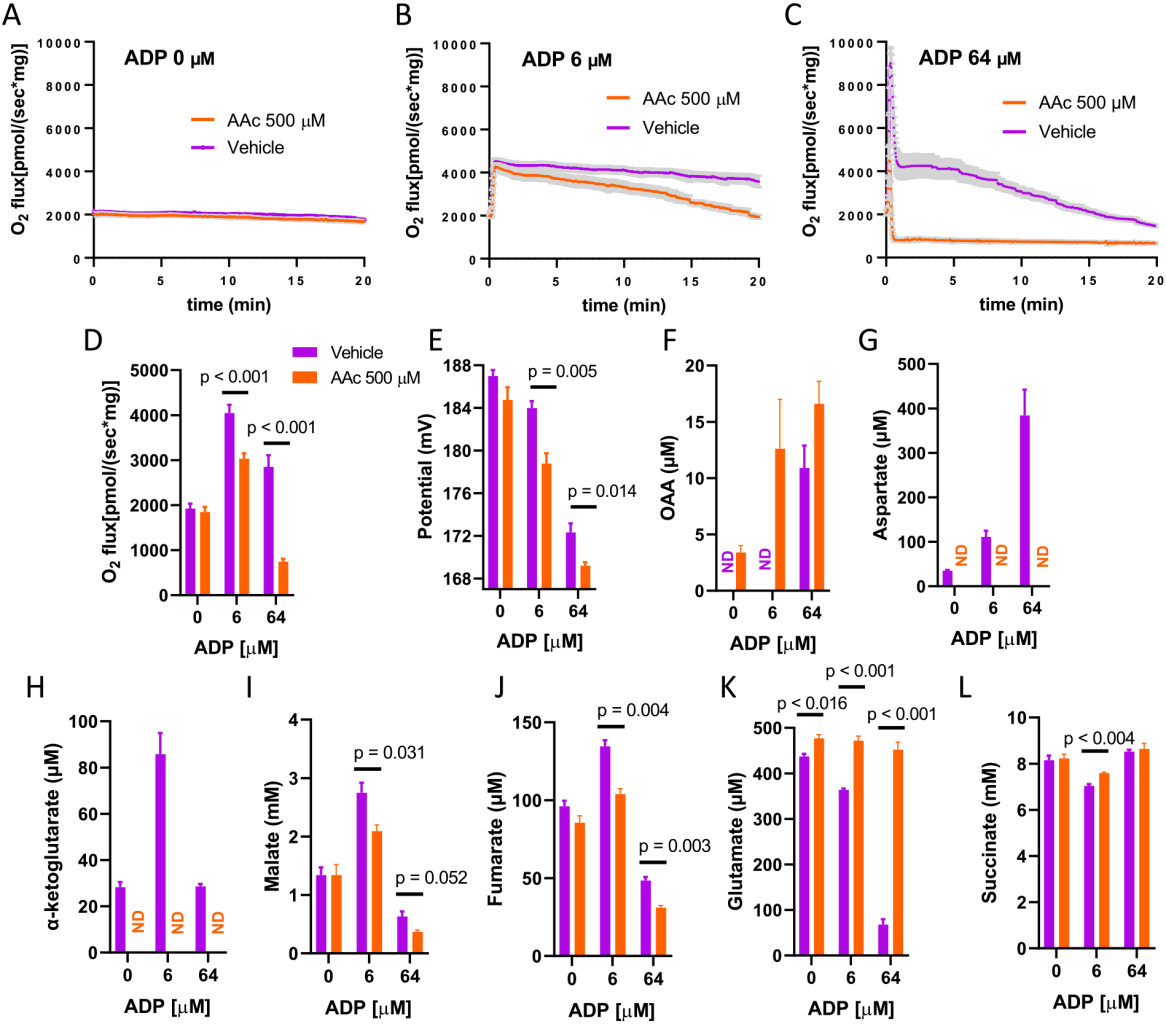
Effect of the transaminase inhibitor, aminooxyacetic acid (AAc) on O2 flux and ΔΨ in muscle mitochondria incubated for 20 min in the absence of ADP or in the presence of clamped concentrations of ADP chosen to induce an increase (6 μM) or decrease (64 μM) in respiration based on the data for incremental administration in Fig. 2. Mitochondria were energized by uniformly 13Clabeled succinate (10 mM) and 13C-glutamate (0.5 mM). A–C) Mean O2 flux (± sem, shaded area) as a function of time at 0, 6, and 64 μM ADP in the presence of vehicle or AAc. D–E) Time averaged O2 flux and inner membrane potential over the incubations shown in panels A to C. *n* = 7 for O2 flux, *n* = 4 for potential. F–L) Metabolite concentrations determined by NMR spectroscopy on disrupted mitochondria in respiratory medium obtained at the conclusion of the 20 min incubations (*n* = 3). ND = not detected. Data represent mean ± sem for all data points. Data were analyzed by 2-way ANOVA with Sidak's method for multiple comparisons.

Fig. 3A-C depict O_2_ flux as a function of time in incubations carried out for 20 min in the presence or absence of AAc at low, mid, or high [ADP] added to modulate ΔΨ. O_2_ flux is constant in the absence of ADP (Fig. 3A) and AAc has no effect. As ADP is incremented to mid-range, ADP induces a sharp rise in respiration which is largely maintained in the absence of AAc but gradually decreases in the presence of AAc (Fig. 3B). At high [ADP], respiration rises sharply after ADP addition with or without AAc but quickly drops (Fig. 3C). However, O_2_ flux drops less and declines more gradually in the absence of AAc, while in the presence of AAc, O_2_ flux drops markedly and to a much lower level. Fig. 3D and E show the average ± sem values for O_2_ flux and ΔΨ over the 20-min incubation periods depicted in Fig. 3A-C, documenting the decrease in ΔΨ according to the concentrations of clamped [ADP]. Fig. 3F to L show the associated changes in metabolite concentrations obtained at the 20-min time points and determined by NMR spectrometry. As shown, higher OAA accumulation (Fig. 3F) corresponds to reduction in O_2_ flux. Aspartate and α-ketoglutarate (α-KG) (Fig. 3G and H), which depend on the transamination reaction (OAA + Glutamate → Aspartate + α-KG), were not detected in the presence of the transaminase inhibitor. The concentrations of α-KG and aspartate at 6 μM ADP in the absence of AAc are similar, as expected from the above reaction. However, a much lower concentration was detected for α-KG than aspartate at 64 μM ADP in the absence of AAc.

The metabolite profiles for malate and fumarate closely match the respiration profiles, as expected based on flux from succinate. The substrate, succinate, deceases most at 6 μM ADP when O_2_ flux peaks, while the substrate, glutamate, in the absence of AAc, decreases in proportion to the level of OAA generated in the incubation. Notably, in the presence AAc, the glutamate level remains the same since AAc inhibits Got2.

**Fig. 4.**
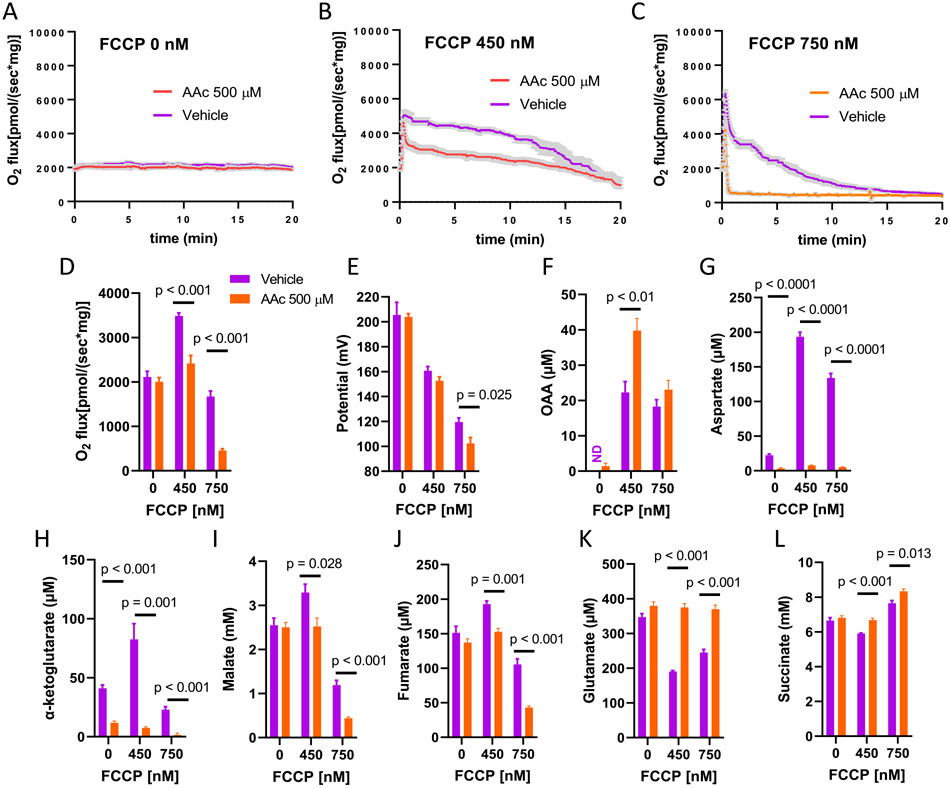
Effect of the transaminase inhibitor, aminooxyacetic acid (AAc) on O2 flux and ΔΨ in muscle mitochondria incubated for 20 min in the absence of FCCP or in the presence of 450 nM or 750 nM FCCP. FCCP concentrations were chosen to induce an increase (450 nM) or decrease (750 nM) in respiration based on preliminary experiments. Mitochondria were energized by uniformly 13C-labeled succinate (10 mM) and 13C-glutamate (0.5 mM). A–C) Mean O2 flux (± sem, shaded area) as a function of time at 0, 450, and 750 nM FCCP in the presence of vehicle or AAc. D–E) Time averaged O2 flux and inner membrane potential over the incubations shown in panels A to C. *n* = 4 for O2 flux and potential. F–L) Metabolite concentrations determined by NMR spectroscopy on disrupted mitochondria in respiratory medium obtained at the conclusion of the 20 min incubations (n = 4). ND = not detected. Data represent mean ± sem for all data points. Data were analyzed by 2-way ANOVA with Sidak's method for multiple comparisons.

The data presented in Fig. 4 are analogous to Fig. 3 but in this case we modulated ΔΨ with FCCP rather than ADP. The amounts of FCCP (0, 450, or 750 nM) were selected based on pilot experiments wherein we incubated mitochondria energized by 10 mM succinate +0.5 mM glutamate with several different concentrations (0 to 750 nM) of FCCP and observed an increase and then a decrease in respiration depending on concentration. The results in Fig. 4 show similar profiles to those in Fig. 3. An exception is that O_2_ flux deteriorated more at the mid-range potential for FCCP titration compared to ADP titration (compare Figs. 3B and 4B). Notably, this difference is associated with detectable OAA in the absence of AAc in Fig. 4F as compared to Fig. 3F.

### 3.4. OAA accumulation over time corresponds to the change in O2 flux

As shown in Figs. 3 and 4, O_2_ flux decreased with time when incubated at lower potential. However, metabolites were only determined at the 20-min time point. To further delineate the relationship between OAA with respiration, we examined metabolite accumulation in muscle mitochondria energized by 10 mM succinate and 0.5 mM glutamate at 450 nM FCCP and incubated for 5 or 20 min (Supplementary Fig. S4). As shown in Supplementary Figs. S4A-S4D, OAA accumulated in proportion to the extent of decrease in respiration and time of incubation and much more so in the presence of AAc. Note that for respiration at mid-range potential for ADP (6 μM) with no AAc (Fig. 3B), there was little drop in respiration over the 20 min incubation and no OAA was detectable (Fig. 3F). This contrasts with the OAA accumulation seen in Fig. 4F at mid-range potential for 450 nM FCCP. Therefore, time-dependent accumulation of OAA explains the difference in respiration profiles when comparing Figs. 3B and 4B. Aspartate accumulation increased with time when incubations were performed without AAc but did not accumulate in the presence of AAc (Fig. S4E).

### 3.5. Metabolite profile and O2 flux in succinate-energized IBAT mitochondria as affected by GOT2-mediated transamination at differing levels of membrane potential

**Fig. 5.**
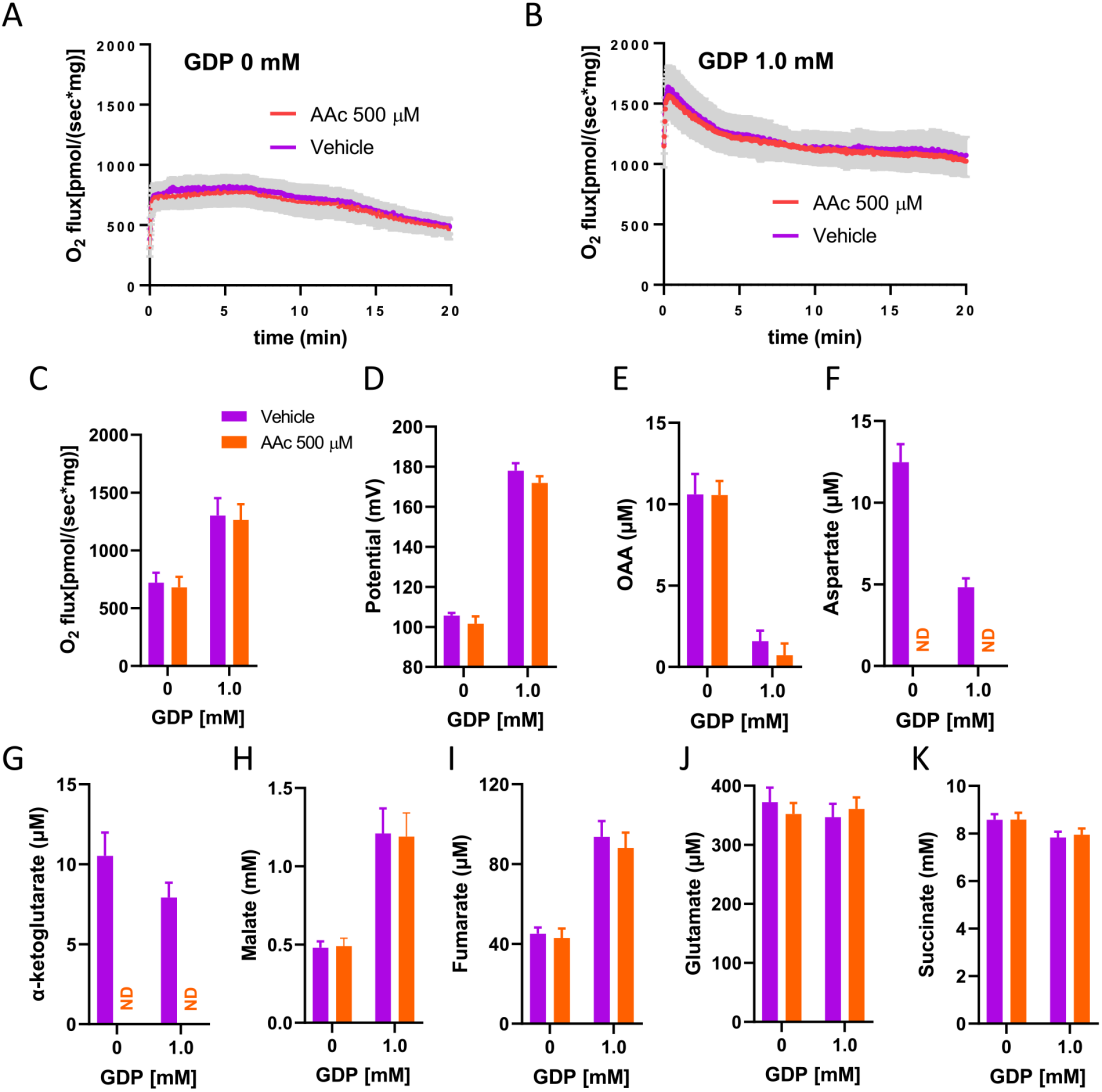
Effect of the transaminase inhibitor, aminooxyacetic acid (AAc) on O2 flux and ΔΨ in IBAT mitochondria incubated for 20 min in the absence or presence of GDP to modulate ΔΨ. Mitochondria were energized by uniformly 13C-labeled succinate (10 mM) and 13C-glutamate (0.5 mM). A–B) Mean O2 flux (± sem, shaded area) as a function of time with or without GDP and in the presence of vehicle or AAc, *n* = 6 for each data point. C–D) Time averaged O2 flux and inner membrane potential over the incubations shown in panels A and B, *n* = 6. E–K) Metabolite concentrations determined by NMR spectroscopy on disrupted mitochondria in respiratory medium obtained at the conclusion of the 20 min incubations, *n* = 5, ND = not detected. Data represent mean ± sem for all data points. Data were analyzed by two-way ANOVA with Sidak's method for multiple comparisons.

We used GDP, a well-recognized potent inhibitor of UCP1 to modulate IBAT ΔΨ, wherein ΔΨ is intrinsically low due to UCP1. As in muscle mitochondria, O_2_ flux in IBAT mitochondria was reduced at lower ΔΨ (without GDP) compared to higher ΔΨ generated by GDP (Fig. 5A and B). We used GDP rather than ADP or FCCP to modulate ΔΨ since we found that these latter compounds had little effect on potential in IBAT mitochondria wherein ΔΨ is already intrinsically low [14,15]. Fig. 5C and D show time-averaged profiles of respiration and potential for IBAT mitochondria incubated with or without GDP and with or without AAc. These profiles differ markedly from those for muscle mitochondria in that the transaminase inhibitor AAc had no detectable effect on O_2_ flux or ΔΨ. Moreover, OAA accumulation was unaffected by the inhibitor (Fig. 5E). AAc did block aspartate formation from OAA (Fig. 5F) and prevent glutamate conversion to α-KG (Fig. 5G). Malate, fumarate, glutamate, and succinate varied as expected with respiration and were unaffected by AAc (Fig. 5H-K).

### 3.6. Tissue dependent metabolism of OAA to pyruvate or aspartate

**Fig. 6.**
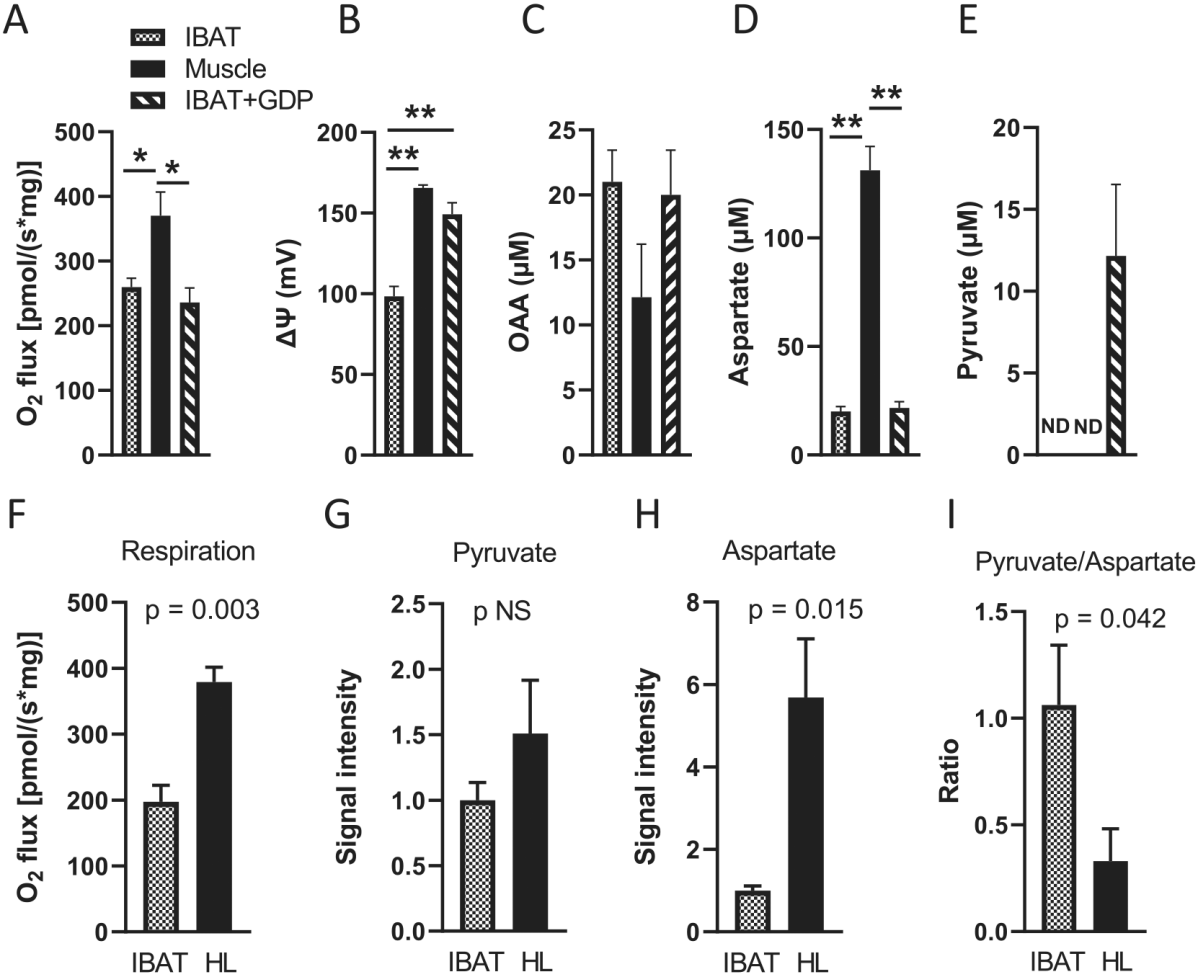
Parameters determined in mitochondria energized by malate to directly drive OAA formation. A–E) O2 flux, ΔΨ, and accumulation of OAA, aspartate, and pyruvate determined by NMR spectroscopy in mitochondria incubated for 20 min with 2 mM uniformly 13C-malate +0.5 mM unlabeled glutamate. IBAT mitochondria were incubated with or without 1 mM GDP. *n* = 4, ND = not detected, * *p* < 0.05, ** *p* < 0.001 by one way ANOVA. F–I) O2 flux, metabolite signal intensities for pyruvate and aspartate (normalized to 1.0 for IBAT), and ratio of pyruvate to aspartate signal intensities determined by LC-MS in IBAT and hindlimb (HL) muscle mitochondria incubated for 20 min with unlabeled malate (5 mM) + unlabeled L-glutamate (0.5 mM) (no added GDP), *n* = 3, *p*-values determined by unpaired, 1-tailed (based on hypothesis generated by prior experiments) t-test. Data for all panels represent mean ± sem. Values for O2 flux and ΔΨ represent average readings over the 20 min incubation times.

Pyruvate was difficult to detect in the above studies of succinate-energized mitochondria, likely because the amounts were too low for our NMR method. To better assess OAA metabolism to pyruvate versus aspartate in muscle and IBAT mitochondria, the organelles were energized by uniformly ^13^C-labeled malate plus a low concertation of unlabeled glutamate and incubated for 20 min (Fig. 6A-E). Under these conditions, respiration is initiated at the malate dehydrogenase step with direct metabolite flow from malate to OAA. Moreover, since respiration is not initiated at SDH, OAA inhibition of SDH and reverse transport from SDH are not relevant. Of further note, only OAA formed from ^13^C-labeled malate would be detected by our 2D NMR method. In these studies, IBAT mitochondria were incubated both with and without 1 mM GDP so that inhibition of UCP1 in IBAT mitochondria would create roughly equal ΔΨ values between IBAT and muscle mitochondria. As expected OAA accumulated in mitochondria of both tissues (Fig. 6C). Aspartate was detected to a far greater extent in muscle mitochondria (Fig. 6D). Pyruvate was not detectable in muscle but detected in IBAT mitochondria incubated in the presence of GDP (Fig. 6E).

To further assess the tissue differences in metabolite flow and to assess pyruvate production with more sensitivity, we performed LC-MS studies. Fig. 6F-I show the results of experiments wherein mitochondria were incubated with unlabeled malate (5 mM) plus unlabeled glutamate (0.5 mM) without GDP. A greater malate concentration was used in panels F to I compared to panels A to E due to the much lower cost of unlabeled versus labeled malate and the higher concentration was felt desirable to provide greater forward drive to OAA by malate dehydrogenase. As shown in Fig. 6F-H, O_2_ flux (Fig. 6F) was greater in hindlimb than IBAT mitochondria associated with greater production of pyruvate and aspartate (Fig. 6G and H). However, we again observed a greater ratio of pyruvate to aspartate for IBAT versus muscle mitochondria (Fig. 6I). Unfortunately, as started above, OAA is too unstable for detection by MS. It should be noted that the accuracy of comparing signal intensities between different mitochondria preparations can be questioned. However, comparing the ratio of signals for aspartate and pyruvate within given preparations should be more robust.

**Fig. 7.**
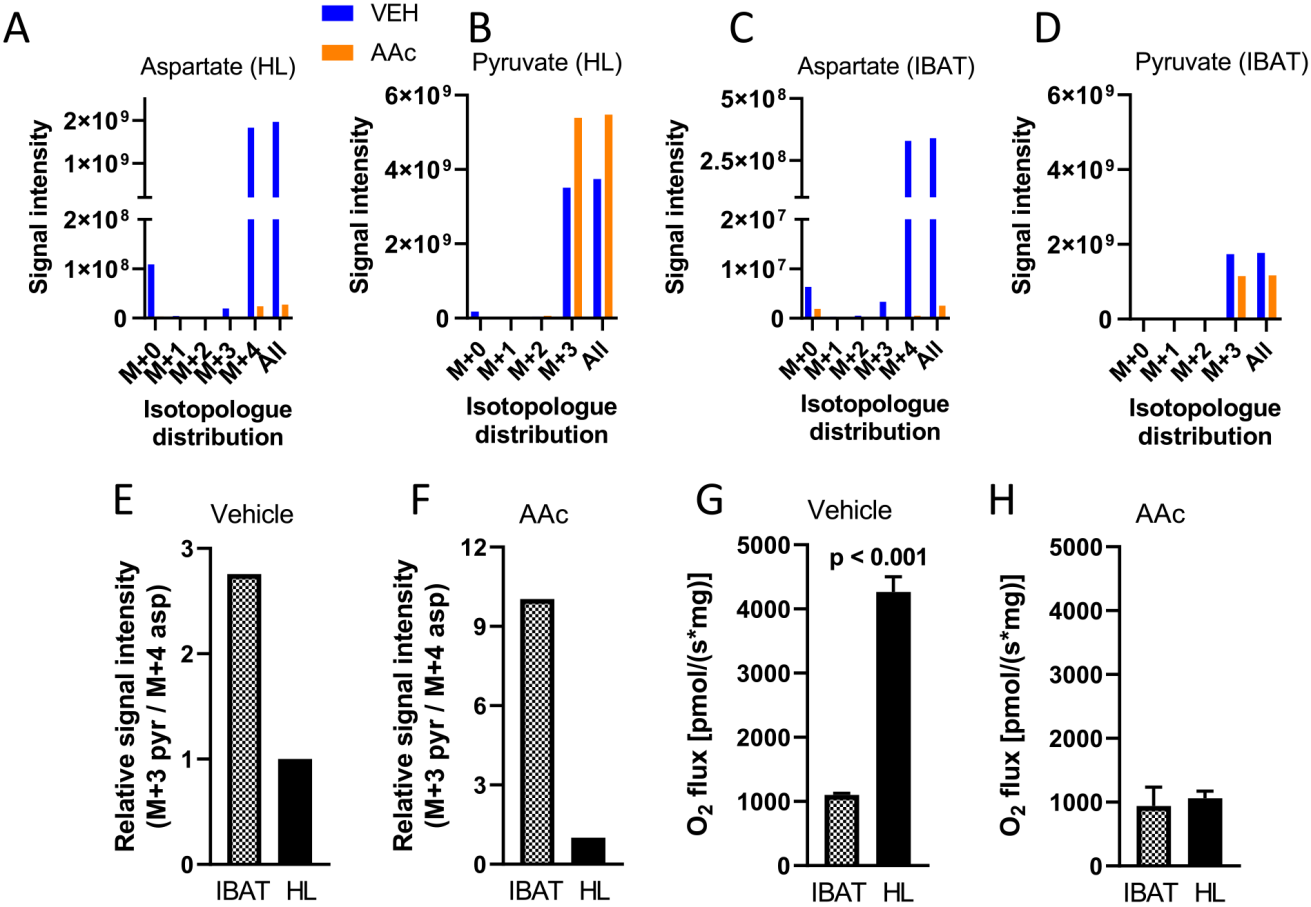
^13^C isotopomer flux analysis and metabolite signal intensities in IBAT and hindlimb (HL) muscle mitochondria incubated for 20 min energized by [U-13C]-succinate (10 mM) + unlabeled Lglutamate (0.5 mM) in the presence or absence of the transaminase inhibitor AAc as determined by LC-MS. Muscle mitochondria were incubated in the presence of a high (64 μM) concentration of ADP, so that both muscle and IBAT (wherein ΔΨ is intrinsically low) were at low potential. A–D) Isotopologue distribution and signal intensities for aspartate and pyruvate in hindlimb (HL) (A and B) and IBAT (C and D) mitochondria. Note the difference in y-axis values (panel A versus panel C) for aspartate. E–F) Ratio of signal intensities (normalized to 1.0 for HL) for the predominant isotopologues (M + 3 pyruvate/M + 4 aspartate) for IBAT versus HL mitochondria in the absence of AAc (panel E) and in the presence of AAc (panel F). G–H) O2 flux in mitochondria incubated in the absence or presence of AAc, *n* = 4, *p*-values determined by unpaired two-tailed *t*-test. The metabolite data (panels A–F) derive from LC-MS analyses of the four combined mitochondrial samples of panels G and H.

In addition, we carried out flux analyses by ^13^C-isotopologue enrichment following incubation with uniformly labeled [U-^13^C]-succinate (10 mM) + unlabeled glutamate (0.5 mM) (Fig. 7). Muscle mitochondria were incubated in the presence of a high (64 μM) concentration of ADP, so that both muscle and IBAT mitochondria (wherein ΔΨ is intrinsically low) were at low potential. As anticipated, aspartate and pyruvate were largely present as M + 4 aspartate and M + 3 pyruvate (Fig. 7A-D). Signal intensities for both metabolites were lower in IBAT compared to hindlimb mitochondria, more so for aspartate (note differences in the Y-axis scales for Fig. 7A versus 7C). However, as shown in Fig. 7E, the ratio of pyruvate to aspartate was approximately 3-fold greater for IBAT mitochondria. Moreover, when performed after incubations with AAc, the ratio differed by about 10-fold (Fig. 7F). Hence, these data are consistent with the NMR and LC-MS findings of Fig. 6.

### 3.7. Tissue and mitochondrial enzyme expression

**Fig. 8.**
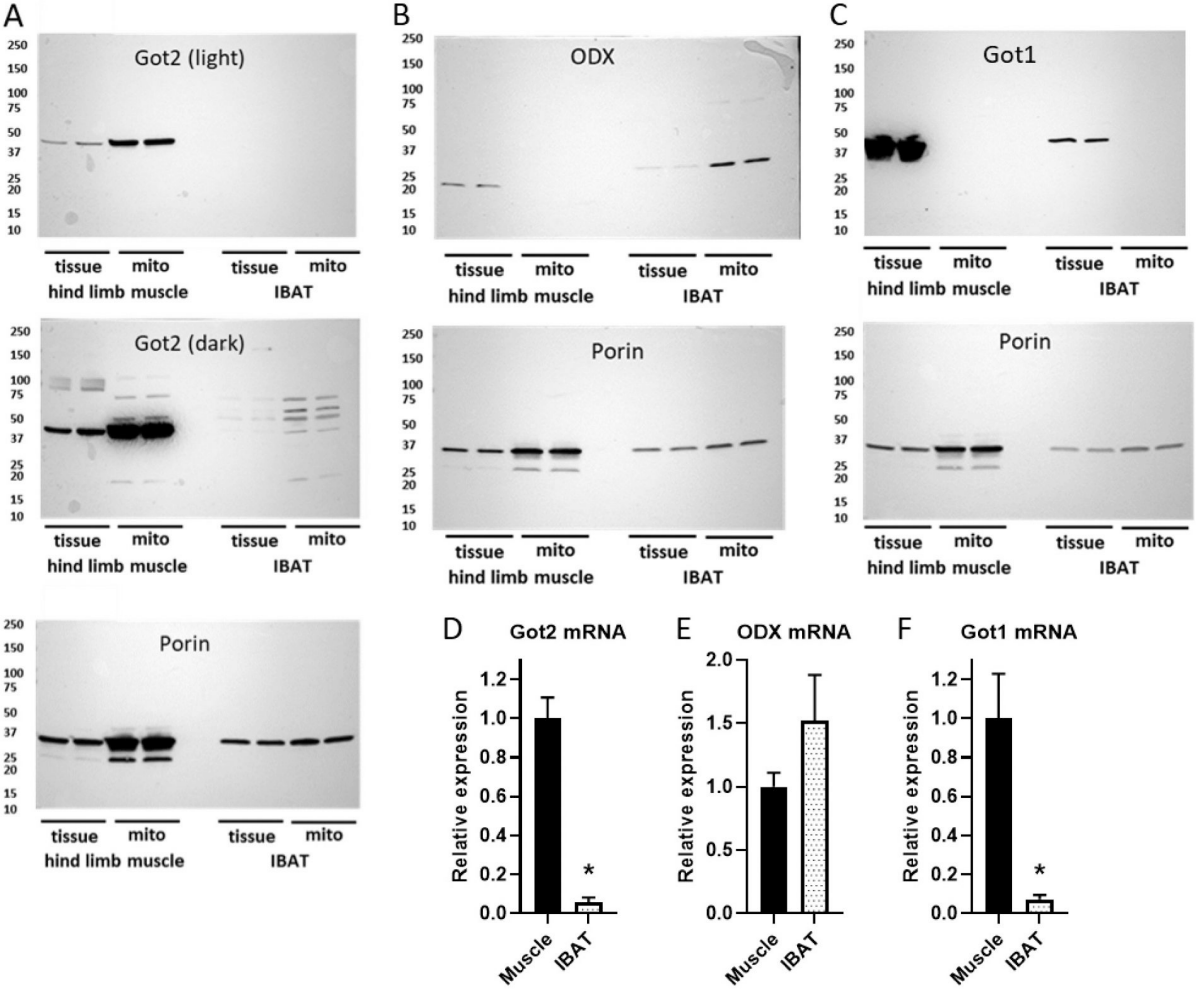
Protein and mRNA expression of mitochondrial Got2 and ODX and the cytoplasmic protein Got1 in skeletal muscle hindlimb and IBAT tissue and isolated mitochondria (mito). A) Immunoblots showing light and dark enhanced chemiluminescence exposures for Got2 and Porin (as a control) on the same blot after destaining. B) ODX and porin after destaining. C) Got 1 and porin after destaining. All images are representative of immunoblots repeated 4 times for ODX, 3 times for Got2, and 2 times for Got1 on mitochondrial and whole tissue isolates. D–F) mRNA expression (relative to mean of 1.0 for muscle) of Got2, Got1, and ODX in skeletal muscle and IBAT tissue, *n* = 3 for all determinations * *p* < 0.05 by 2-tailed, unpaired ttest.

At the protein level, the mitochondrial form of transaminase (Got2) was clearly expressed in muscle mitochondria while there was only questionable, if any, expression in IBAT mitochondria (Fig. 8A, light and dark exposures). On the other hand, the ODX enzyme was detected only in IBAT mitochondria (Fig. 8B). The correct band size for ODX is ~30 KDa. We think that the lower bands observed in muscle tissue (not seen in muscle mitochondria) are non-specific. For perspective, we also examined the expression of cytoplasmic transaminase Got1, which we observed in both muscle and IBAT tissue (but more so in muscle). However, Got1 was not detectable in mitochondria of either tissue (Fig. 8C). At the mRNA level (Fig. 8D-F), Got2 and Got1 were expressed to a much greater extent in muscle than IBAT, while ODX mRNA was expressed in both tissues, but more so in IBAT (although not statistically different).

The presence of M + 3 labeled pyruvate in muscle (Fig. 7B) despite undetectable ODX (Fig. 8) suggests that pyruvate may have been derived from malate. There are two forms of mitochondrial malic enzyme, ME2 and ME3 [34,35]. Therefore, we also examined the expression of these proteins in IBAT and muscle as well as in brain as a positive control wherein the enzyme is abundant [34]. As shown in Supplemental Fig. S5, these proteins, although abundant in brain mitochondria, were far less expressed (ME3) or barely expressed at all (ME2) in IBAT and muscle mitochondria. If anything, ME3 was slightly more detectable in muscle and does not explain the greater pyruvate to aspartate ratio in IBAT versus muscle. But the expression in muscle could explain the pyruvate observed in Fig. 7.

## 4. Discussion

Our past findings show that OAA inhibition of SDH regulates complex II respiration in a ΔΨ-dependent manner for both skeletal muscle and IBAT mitochondria. Here, we questioned how mitochondrial OAA might be further metabolized and how mitochondrial metabolism of OAA might affect respiration as well as whether these effects would depend on ΔΨ and tissue type.

First, we showed that both glutamate and pyruvate, when administered to succinate-energized skeletal muscle mitochondria under phosphorylating conditions (low ΔΨ, ADP at 32 μM), increased respiration in a dose-dependent fashion and to approximately the same extent (Fig. 1A). A low dose of glutamate was added to drive OAA transamination to aspartate while a low amount of pyruvate was added to generate acetyl-CoA to drive OAA to citrate. Neither glutamate nor pyruvate, when added alone, had more than minimal effects on O_2_ flux. When the same studies were done in IBAT mitochondria, the results were different. Although pyruvate again markedly increased O_2_ flux (Fig. 1B), glutamate had little effect, suggesting that IBAT has far less capacity to clear OAA via Got2.

These observations led us to investigate the role of transaminase inhibition. As shown in Fig. 2A, titrating succinate-energized muscle mitochondria with ADP initially increased then decreased O_2_ flux, consistent with our past findings [16]. However, as shown in Fig. 2B, different results were observed when the same experiment was performed with addition of a low dose of glutamate to enable transamination. In the absence of the transaminase inhibitor AAc, glutamate reduced the drop in respiration at lower ΔΨ (i.e., higher ADP) as expected. However, in the presence of AAc, the downward turn in respiration was far more marked, implying that preventing transamination by AAc led to OAA accumulation resulting in inhibition of SDH and, hence, O_2_ flux.

To directly measure the concentrations of OAA and other TCA intermediates and to assess the effect of OAA metabolism by Got2 in skeletal muscle, we carried out metabolite studies by NMR for mitochondrial samples after 20 min of incubation in the presence or absence of AAc (Fig. 3). It is important to remember that our 2D NMR method only detects ^13^C-labeled compounds derived from the uniformly ^13^C-labeled substrates (succinate and glutamate in this experiment). Because O_2_ flux is ΔΨ-dependent, we carried out incubations under conditions of high potential (state 4, no ADP added), mid-range potential (6 μM ADP, higher respiration), and low potential (64 μM ADP, low respiration). O_2_ flux was altered as predicted based on ΔΨ (Fig. 3A-D). Metabolite data (Fig. 3F) showed that OAA increased as expected at low potential (or high ADP). However, with transaminase inhibition, OAA began to increase even at mid-range potential and increased to higher levels at low ΔΨ. Given that OAA inhibits SDH, these transaminase-dependent changes in OAA are in line with the observed difference in respiration (3A-3D). The effectiveness of transaminase inhibition is documented by the absence of detectable aspartate and α-KG in presence of AAc (Fig. 3G-H). Changes in malate and fumarate were as expected based on respiration initiated at complex II, while glutamate and succinate concentrations reflect consumption of the substrates. Overall, these findings show that inhibition of Got2 by AAc led to a ΔΨ-dependent increase in OAA and a ΔΨ-dependent decrease in muscle mitochondrial respiration.

We observed a high concentration of αKG at 6 μM ADP in the absence of AAc (Fig. 3H). This is not surprising as it would result from GOT2-catalyzed glutamate and OAA conversion to aKG and aspartate. However, it is surprising that, at high (64 μM) ADP, αKG was much lower than aspartate in the absence of AAc (Fig. 3G and H). Here we can only speculate. It is of note that glutamate is also low (Fig. 3K) consistent with consumption of the added substrate. Note also that the amount of glutamate consumed (based on Fig. 3K and considering the added concentration of 500 μM) is in rough agreement with the amount of aspartate generated (Fig. 3G) suggesting that the added glutamate is depleted by GOT2-mediated conversion of OAA to aspartate with glutamate conversion to αKG. So, the fact that αKG is low (Fig. 3H) suggests that the compound is further metabolized possibly to succinyl-CoA. Note that if αKG simply exited mitochondria, we would still have measured the labeled compound in the Oxygraph chamber media saved for NMR. However, an issue with conversion to succinyl-CoA is that this would be opposed by the large amount of succinate preventing further metabolism of succinyl-CoA. But also note that the low αKG is observed only at high ADP (low ΔΨ). Under these conditions our past work [14,16,18] has demonstrated that at low potential the NADH/NAD+ ratio is shifted towards greater NAD^+^ which, as co-factor, may drive the αKG dehydrogenase reaction to succinyl-CoA. It is also possible that succinyl-CoA exited mitochondria or simply broke down.

Modulating ΔΨ with ADP additions also increases ATP production which might, by itself, alter metabolite flux. Therefore, we also determined the effect of change in membrane potential by chemical uncoupling using different FCCP concentrations. As shown (Fig. 4), the data for FCCP perturbation are nearly identical to those for ADP titration. One explainable difference regards the concentration of OAA at mid-range potential. OAA, in the absence of AAc, is present at mid-range potential for FCCP (450 nM) but not for mid-range potential for ADP (compare Figs. 3F and 4F). We believe, the reason for this is that 450 nM FCCP is somewhat more potent at reducing potential than 6 μM ADP. Consequently, respiration drops over time at mid-range FCCP (Fig. 4B), but barely drops at all at mid-range ΔΨ when modulated by ADP (Fig. 3B). This suggested a time course effect of OAA accumulation over the 20-min incubation conditions. To further address this, we examined OAA production after incubation at FCCP 450 nM for 5 min as well as 20 min (Fig. S4). We observed that in the absence of AAc, OAA only accumulated to a detectable extent after the 20 min incubation time (Fig. S4D). However, OAA did begin to accumulate at 5 min in the presence of AAc (Fig. S4D), leading to far greater drop in the early part of the O_2_ flux profile (compare figs. S4A and S4B).

In further experiments, we examined metabolite accumulation and O_2_ flux under differing levels of potential in IBAT, as opposed to muscle, mitochondria (Fig. 5). Here, we used GDP to raise potential over the low level of ΔΨ intrinsic to IBAT. As in the case of muscle, respiration was lower at lower potential (without GDP) (Fig. 5A-C). However, for IBAT mitochondria, transaminase inhibition had no effect on either ΔΨ or OAA (Fig. 5D and E). This was the case at either level of membrane potential (GDP 0 or 1.0 mM). Again, the effect of AAc to inhibit transaminase was documented based on the complete absence of detectable aspartate and αKG (Fig. 5F and G). AAc also did not alter malate, fumarate, glutamate, or succinate consistent with no effect on respiration. We further note that in the absence of AAc, IBAT mitochondria at low ΔΨ (without GDP) produced only ~12.5 μM aspartate (Fig. 5F) versus ~380 μM aspartate generated from muscle mitochondria also at low ΔΨ (with 64 μM ADP) (Fig. 3G). These results indicate a much lower capacity in IBAT mitochondria to convert OAA to aspartate than muscle mitochondria. This is consistent with our mRNA profiling and immunoblot assays which revealed that Got2 is clearly expressed in muscle mitochondria, while very little Got2 is detectable in IBAT mitochondria (Fig. 8A and D). Of note, the same trend was detected for cytoplasmic Got1 (Fig. 8C). Moreover, we detected ODX in IBAT but not muscle mitochondria (Fig. 8B). These data are consistent with differential metabolism of OAA in muscle versus IBAT mitochondria and suggest that OAA in IBAT mitochondria might have undergone metabolism in another fashion, possible to pyruvate by oxaloacetate decarboxylase (ODX) or to citrate by reaction with acetyl-CoA. Unfortunately, we could not detect pyruvate by NMR under these conditions. Acetyl-CoA was not added but could have formed from pyruvate generated by ODX.

To detect OAA metabolism to pyruvate is difficult by NMR since the amount of pyruvate is too low for this method. To boost sensitivity, we used [U-^13^C]-malate plus a low concentration of unlabeled glutamate as substrates in the incubation of muscle and IBAT mitochondria (Figs. 6A-E). Under these conditions, respiration starts at the malate dehydrogenase step which is not subjected to OAA feedback inhibition of SDH and drives metabolite flow directly to OAA, hence, potentially generating more pyruvate. By incubating mitochondria in this way, we observed that pyruvate was only detected in IBAT mitochondria, at least in the presence of GDP. In contrast, aspartate formation was far greater in muscle that IBAT mitochondria. These results are consistent with the differential expression of Got2 and ODX between these tissues (Fig. 8). At present, we do not know why GDP was needed for detection of OAA in IBAT mitochondria and can only speculate. Possibilities, well beyond the scope of this report, include the effect of ΔΨ to alter the transport of malate or interaction of malate dehydrogenase with other proteins [36], the effect of ΔΨ on the NADH/NAD+ ratio [37], or other action that might increase flux through OAA.

Using LC-MS, we were better able to detect pyruvate production. As shown in Fig. 6F to 6I, the greater flow towards pyruvate in IBAT was further confirmed by incubation of mitochondria energized by unlabeled malate. Moreover, incubations performed using [U—^13^C]-succinate to energize complex II further documented the tissue difference in OAA flow to pyruvate as opposed to aspartate (Fig. 7). Under these conditions, M + 3 pyruvate and M + 4 aspartate were, by far, the predominate isotopologues indicating direct production from the uniformly ^13^C-labeled succinate (through OAA) and indicating a considerably greater pyruvate to aspartate ratio in IBAT mitochondria versus muscle (Fig. 7E). This ratio was further increased with transaminase inhibition (Fig. 7F) which should increase back pressure towards OAA clearance to pyruvate. Note that although OAA is too unstable for MS detection, these uniformly labeled products (M + 3 pyruvate and M + 4 aspartate) could only have been generated by fully ^13^C-labeled OAA from the added succinate.

Taken together, our findings strongly support a ΔΨ-dependent and tissue-dependent role for mitochondrial transamination in regulating respiration in muscle mitochondria and at least suggest a role of ODX to regulate respiration in IBAT mitochondria. It is true that blocking OAA conversion to aspartate should not depend on ΔΨ. However, the amount of OAA produced does depend on ΔΨ. Relative to respiration, the amount of OAA formed at lower ΔΨ is greater than the amount at higher ΔΨ, adding to the total amount of OAA available for inhibition of SDH. Note that, as seen in Figs. 3D and 4D, the inhibition of respiration by AAc is greater as ΔΨ is lowered, consistent with our hypothesis (see introduction) that “altering the metabolic clearance of OAA would alter complex II-energized mitochondrial respiration and that this would occur in a ΔΨ-dependent fashion”.

**Fig. 9.**
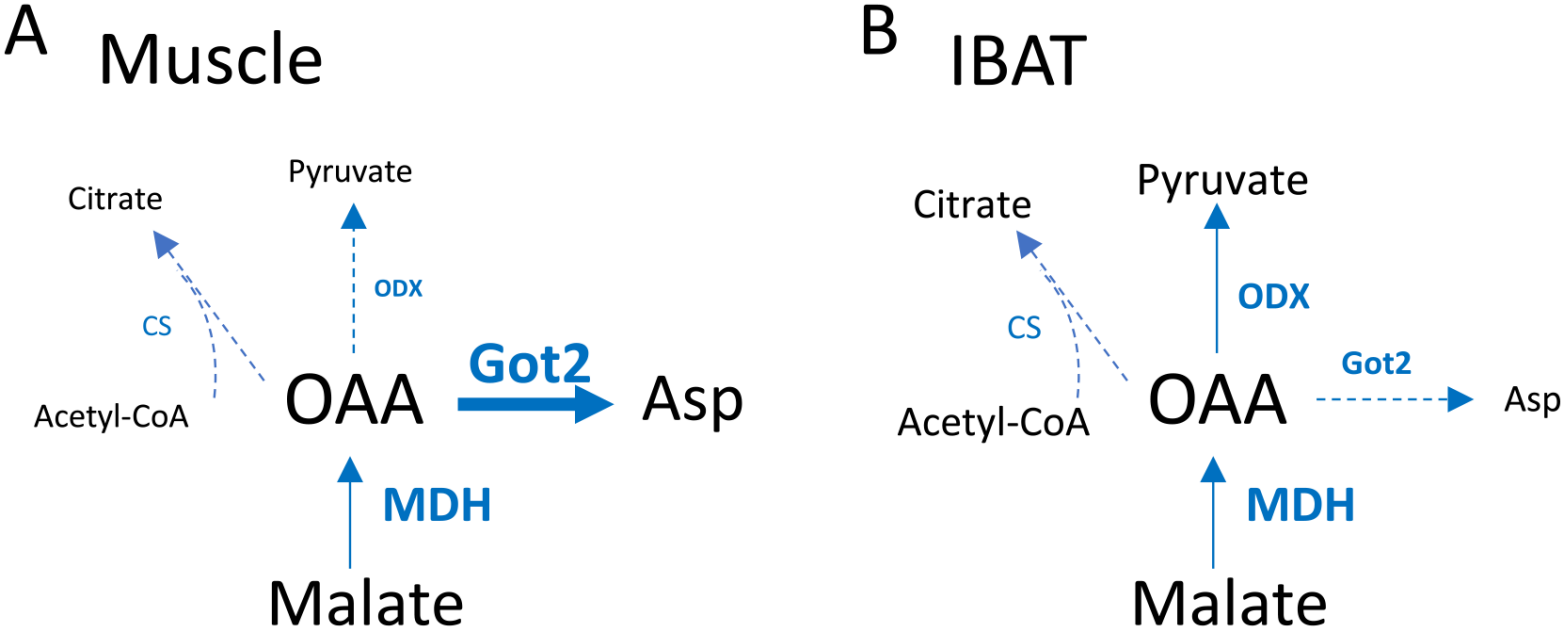
Schematic diagram depicting proposed oxaloacetate (OAA) metabolism in skeletal muscle compared to IBAT mitochondria during complex II-energized respiration. Weight of arrows and text size reflect relative reaction activity. Dotted lines depict less significant (lower flux) or less certain pathways. Abbreviations: Got2, mitochondrial glutamicoxaloacetic transaminase; ODX, oxaloacetate decarboxylase; MDH malate dehydrogenase; OAA oxaloacetate; Asp, aspartate; CS, citrate synthase.

Fig. 9 schematically summarizes the relative complex II-energized flux of OAA in muscle (Fig. 9A) versus IBAT (Fig. 9B) mitochondria. The Got2 reaction is far more active in muscle, while the ODX step is at least mildly active in IBAT with little, if any, in muscle. The citrate synthase reaction depends on acetyl-CoA which is not generated by succinate. However, if ODX is active in IBAT, this might generate a small amount of pyruvate which in turn could produce acetyl CoA.

Although the physiologic significance of OAA regulation of SDH and O_2_ flux still needs further study, we suggest that its relevance is evident in that evolution has enabled a potent inhibitory effect of OAA in a critical metabolic pathway, i.e., the TCA cycle. OAA is known as a ligand or cofactor important in the assembly and interactions of SDH subunits [38,39]. We speculate that OAA can be viewed as critical in balancing flux control by inhibiting SDH while also providing substate for flow through citrate synthase. The exact effect should depend on availability of NADH, acetyl-CoA, and glutamate. Conceivably, OAA inhibition of SDH might serve as a metabolic brake, possibly important in regulating overall energy flow and/or controlling reactive oxygen species (ROS) production known to occur by reverse electron transport from complex II to complex I [40]. ROS produced in this way my be particularly prominent during ischemia/reperfusion [41]. Further, Molinie, et al. recently provided evidence supporting OAA inhibition of SDH as a way to adjust the balance between malate dehydrogenase driven anaplerosis and malate production from succinate [6]. Moreover, there is evidence that SDH is critical in regulating neurologic, oncologic, and cardiovascular function [42-44].

We acknowledge certain limitations. The current study was performed in isolated mitochondria. On the other hand, this type of study is required to examine metabolite flow independent of cytoplasmic reactions that would modulate OAA metabolism. Moreover, the specific effect of initiating substrate flow at specific sites, for example SDH, can be accomplished in these isolated organelles. Preferential clearance of OAA to pyruvate in IBAT versus muscle mitochondria was an incidental finding likely related to ODX expression. Further documentation by ODX inhibition still needs demonstration.

In summary, our major finding is that OAA clearance in muscle mitochondria is dependent on Got2-mediated transamination to aspartate. Inhibition of this reaction translates to reduced respiration. This effect is tissue-dependent and most active at low membrane potential, when OAA inhibition of SDH is most evident. Secondarily, we observed that transamination of OAA is minimally, if at all, operative in IBAT mitochondria wherein we found evidence suggesting some degree of OAA clearance to pyruvate by decarboxylation.

## Supplementary Material

supplement

## Data Availability

Data will be shared upon request by contacting William Sivitz, william-sivitz@uiowa.edu, University of Iowa, Division of Adult Endocrinology and Metabolism, Iowa City IA, 52246, USA.

## Footnotes

1 We use the term ΔΨ to specify inner mitochondrial membrane potential as commonly measured in electrochemical fashion by our Oxygraph respirometer. We make no attempt to differentiate the electrical versus pH-dependent components of the potential gradient referred to as the proton motive force.

## Abbreviations:

2DOG: 2-deoxyglucose
2DOGP: 2-deoxyglucose phosphate
AAc: aminooxy acetic acid
FCCP: carbonyl cyanide p-trifluoromethoxy-phenyl-hydrazone
Got2: glutamic-oxaloacetic transaminase
IBAT: interscapular brown adipose tissue
MDH: malate dehydrogenase
TMPD: *N,N,N′,N′*-Tetramethyl-*p*-phenylenediamine dihydrochloride
OAA: oxaloacetic acid
ODX: oxaloacetate decarboxylase
RET: reverse electron transport
ROS: reactive oxygen species
SDH: succinate dehydrogenase
α-KG: α-ketoglutarate
ΔΨ: membrane potential
LC-MS: liquid chromatography mass spectrometry
ME2: malic enzyme 2
ME3: malic enzyme 3
