# Supplementary material for: Metabolic clearance of oxaloacetate and mitochondrial complex II respiration: Divergent control in skeletal muscle and brown adipose tissue
Source: Biochim Biophys Acta Bioenerg. Author manuscript; Available in PMC 2023 May 28. (PMC10225247; doi:10.1016/j.bbabio.2022.148930)
Supplement: supplement [file NIHMS1898125-supplement-supplement.pptx]

## Slide 1
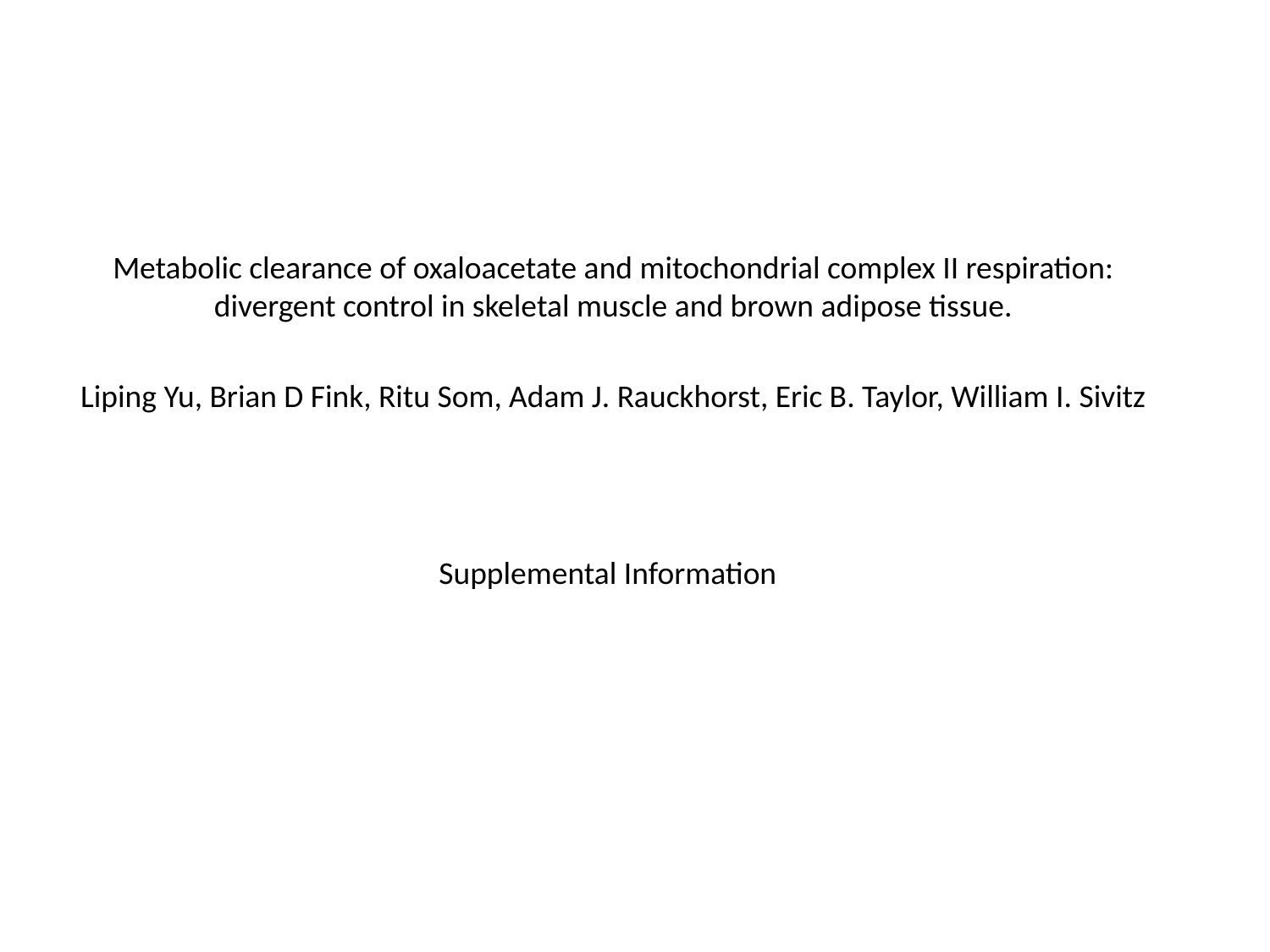

Metabolic clearance of oxaloacetate and mitochondrial complex II respiration: divergent control in skeletal muscle and brown adipose tissue.
Liping Yu, Brian D Fink, Ritu Som, Adam J. Rauckhorst, Eric B. Taylor, William I. Sivitz
Supplemental Information

## Slide 2
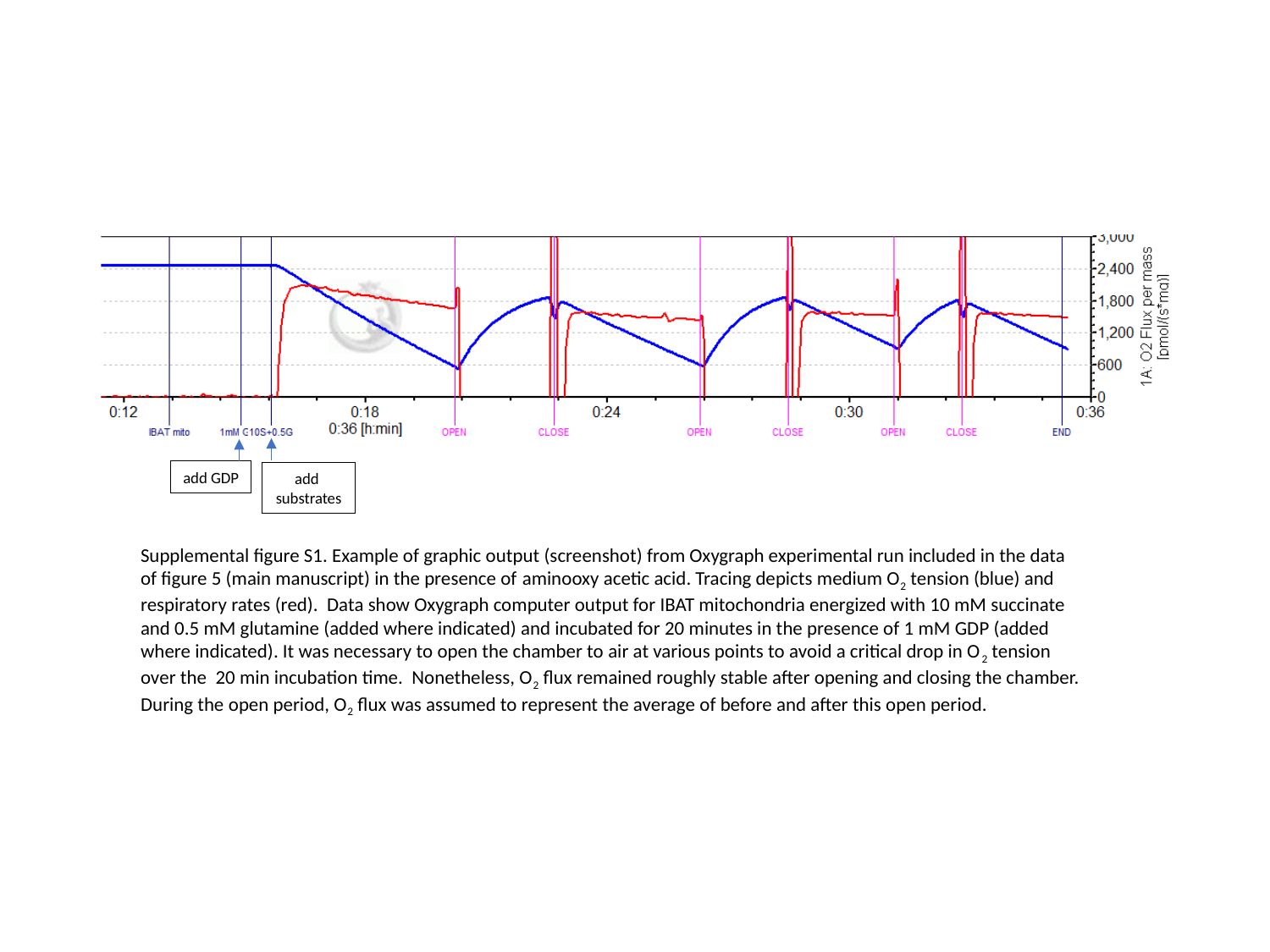

add GDP
add
substrates
Supplemental figure S1. Example of graphic output (screenshot) from Oxygraph experimental run included in the data of figure 5 (main manuscript) in the presence of aminooxy acetic acid. Tracing depicts medium O2 tension (blue) and respiratory rates (red). Data show Oxygraph computer output for IBAT mitochondria energized with 10 mM succinate and 0.5 mM glutamine (added where indicated) and incubated for 20 minutes in the presence of 1 mM GDP (added where indicated). It was necessary to open the chamber to air at various points to avoid a critical drop in O2 tension over the 20 min incubation time. Nonetheless, O2 flux remained roughly stable after opening and closing the chamber. During the open period, O2 flux was assumed to represent the average of before and after this open period.

## Slide 3
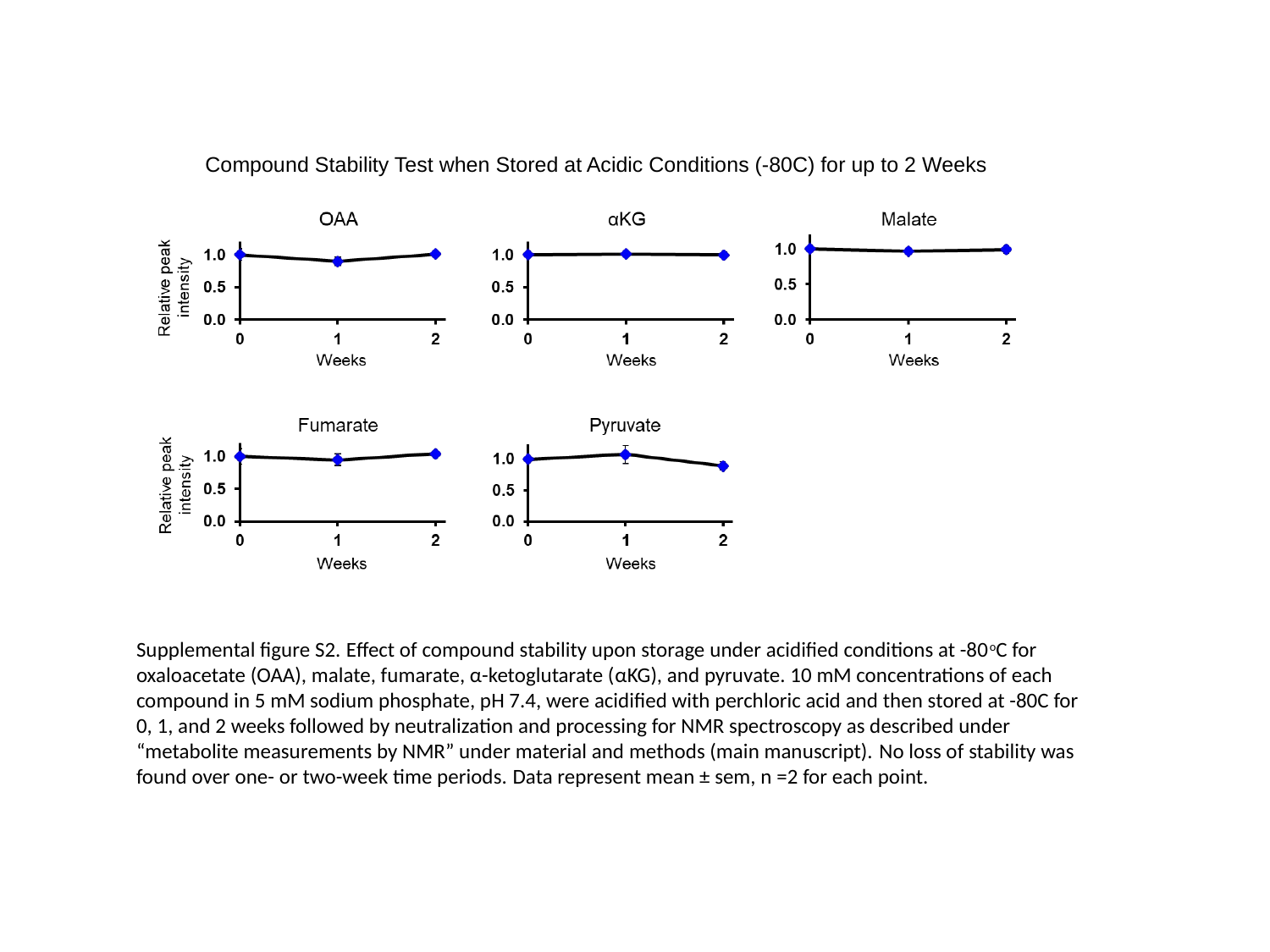

Compound Stability Test when Stored at Acidic Conditions (-80C) for up to 2 Weeks
Supplemental figure S2. Effect of compound stability upon storage under acidified conditions at -80oC for oxaloacetate (OAA), malate, fumarate, α-ketoglutarate (αKG), and pyruvate. 10 mM concentrations of each compound in 5 mM sodium phosphate, pH 7.4, were acidified with perchloric acid and then stored at -80C for 0, 1, and 2 weeks followed by neutralization and processing for NMR spectroscopy as described under “metabolite measurements by NMR” under material and methods (main manuscript). No loss of stability was found over one- or two-week time periods. Data represent mean ± sem, n =2 for each point.

## Slide 4
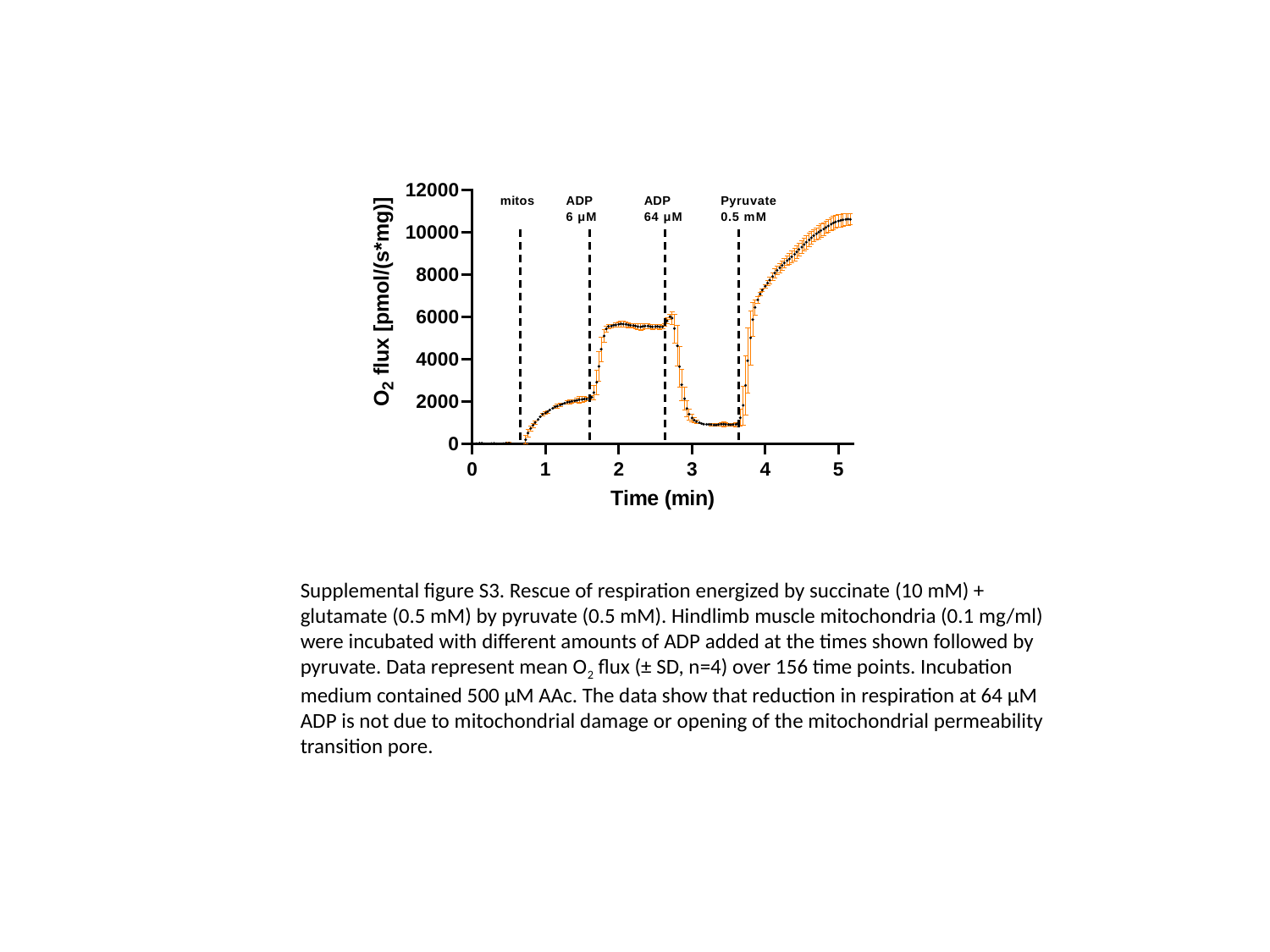

Supplemental figure S3. Rescue of respiration energized by succinate (10 mM) + glutamate (0.5 mM) by pyruvate (0.5 mM). Hindlimb muscle mitochondria (0.1 mg/ml) were incubated with different amounts of ADP added at the times shown followed by pyruvate. Data represent mean O2 flux (± SD, n=4) over 156 time points. Incubation medium contained 500 µM AAc. The data show that reduction in respiration at 64 µM ADP is not due to mitochondrial damage or opening of the mitochondrial permeability transition pore.

## Slide 5
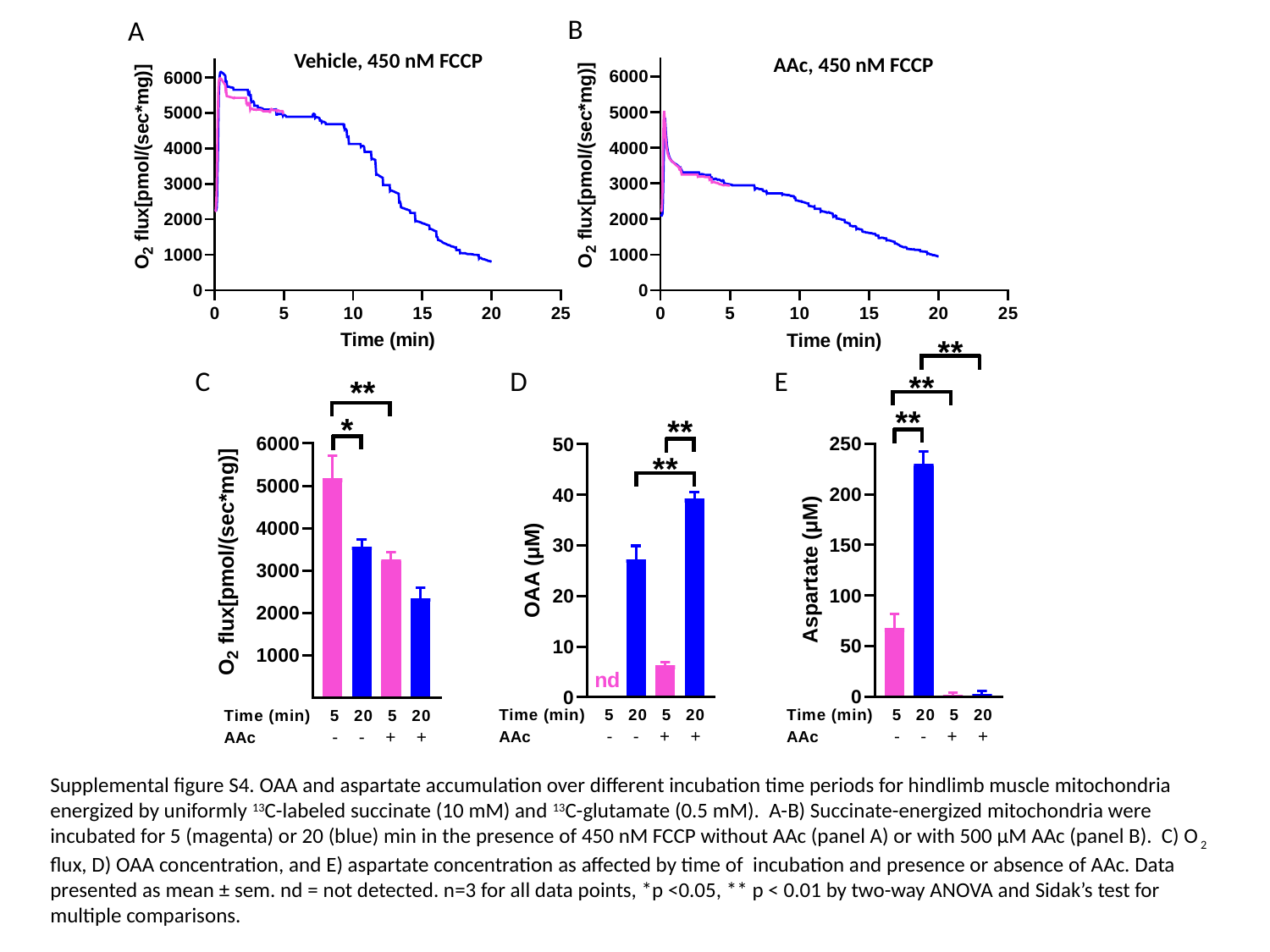

B
A
Vehicle, 450 nM FCCP
AAc, 450 nM FCCP
C
D
E
Supplemental figure S4. OAA and aspartate accumulation over different incubation time periods for hindlimb muscle mitochondria energized by uniformly 13C-labeled succinate (10 mM) and 13C-glutamate (0.5 mM). A-B) Succinate-energized mitochondria were incubated for 5 (magenta) or 20 (blue) min in the presence of 450 nM FCCP without AAc (panel A) or with 500 µM AAc (panel B). C) O2 flux, D) OAA concentration, and E) aspartate concentration as affected by time of incubation and presence or absence of AAc. Data presented as mean ± sem. nd = not detected. n=3 for all data points, *p <0.05, ** p < 0.01 by two-way ANOVA and Sidak’s test for multiple comparisons.

## Slide 6
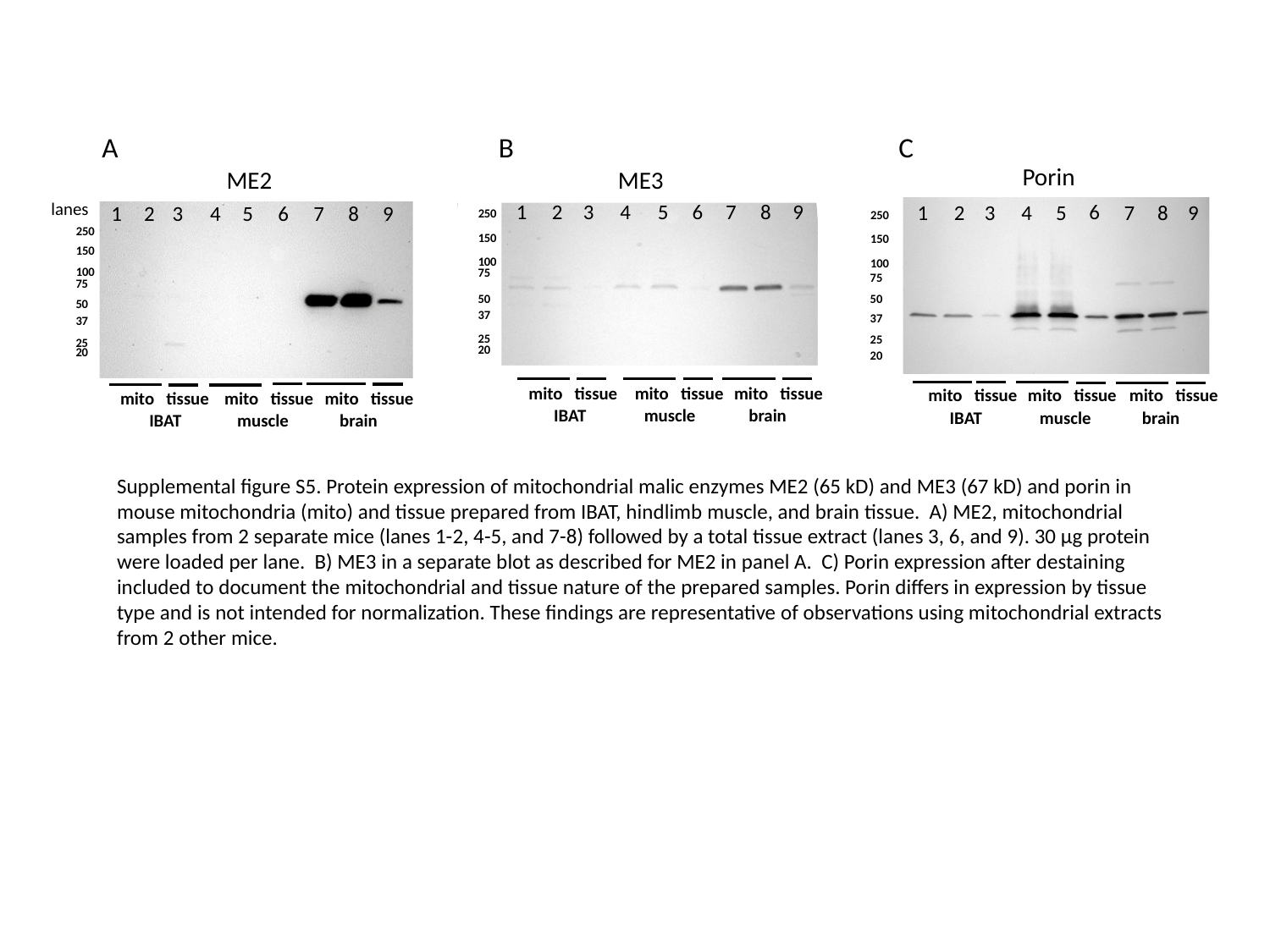

A
B
C
Porin
ME2
lanes
1
2
4
5
6
7
8
9
3
250
150
100
75
50
37
25
20
mito tissue
mito tissue
mito tissue
muscle
IBAT
brain
ME3
1
2
3
4
5
6
7
8
9
250
150
100
75
50
37
25
20
mito tissue
mito tissue
mito tissue
muscle
IBAT
brain
6
1
2
3
4
5
8
9
7
250
150
100
75
50
37
25
20
mito tissue
mito tissue
mito tissue
muscle
IBAT
brain
Supplemental figure S5. Protein expression of mitochondrial malic enzymes ME2 (65 kD) and ME3 (67 kD) and porin in mouse mitochondria (mito) and tissue prepared from IBAT, hindlimb muscle, and brain tissue. A) ME2, mitochondrial samples from 2 separate mice (lanes 1-2, 4-5, and 7-8) followed by a total tissue extract (lanes 3, 6, and 9). 30 µg protein were loaded per lane. B) ME3 in a separate blot as described for ME2 in panel A. C) Porin expression after destaining included to document the mitochondrial and tissue nature of the prepared samples. Porin differs in expression by tissue type and is not intended for normalization. These findings are representative of observations using mitochondrial extracts from 2 other mice.

## Slide 7
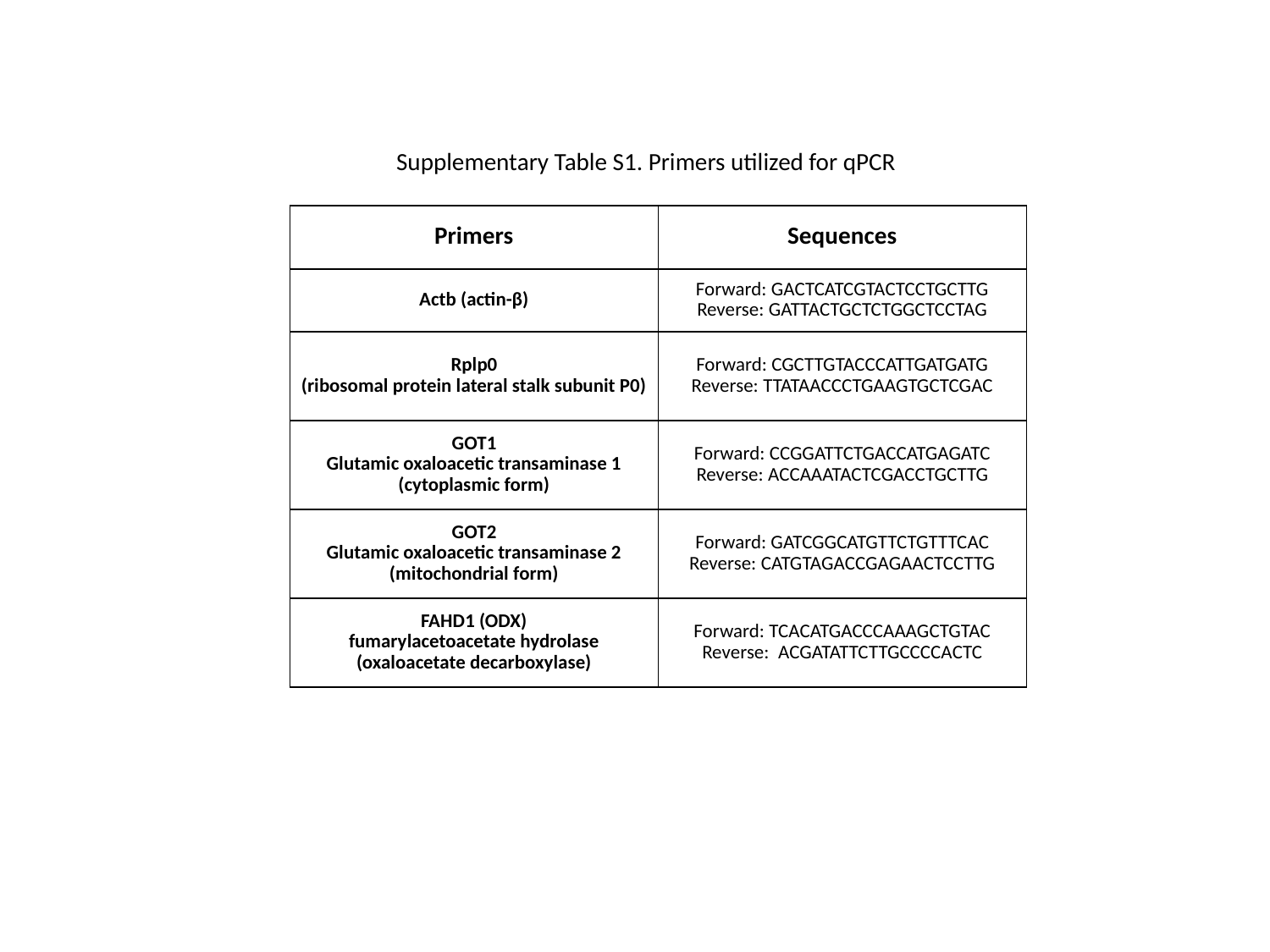

Supplementary Table S1. Primers utilized for qPCR
| Primers | Sequences |
| --- | --- |
| Actb (actin-β) | Forward: GACTCATCGTACTCCTGCTTG Reverse: GATTACTGCTCTGGCTCCTAG |
| Rplp0 (ribosomal protein lateral stalk subunit P0) | Forward: CGCTTGTACCCATTGATGATG Reverse: TTATAACCCTGAAGTGCTCGAC |
| GOT1 Glutamic oxaloacetic transaminase 1 (cytoplasmic form) | Forward: CCGGATTCTGACCATGAGATC Reverse: ACCAAATACTCGACCTGCTTG |
| GOT2 Glutamic oxaloacetic transaminase 2 (mitochondrial form) | Forward: GATCGGCATGTTCTGTTTCAC Reverse: CATGTAGACCGAGAACTCCTTG |
| FAHD1 (ODX) fumarylacetoacetate hydrolase (oxaloacetate decarboxylase) | Forward: TCACATGACCCAAAGCTGTAC Reverse: ACGATATTCTTGCCCCACTC |
